# Is gene expression among women with rheumatoid arthritis dysregulated during a postpartum flare?

**DOI:** 10.1186/s13075-021-02418-w

**Published:** 2021-01-18

**Authors:** Matthew Wright, Mette K. Smed, J. Lee Nelson, Jørn Olsen, Merete L. Hetland, Vibeke Zoffmann, Damini Jawaheer

**Affiliations:** 1 Children’s Hospital Oakland Research Institute, UCSF Benioff Children’s Hospital Oakland, 5700 Martin Luther King Jr. Way, Oakland, CA 94609 USA; 2 Juliane Marie Center, Copenhagen University Hospital, Rigshospitalet, Copenhagen, Denmark; 3 Fred Hutchinson Cancer Research Center, Seattle, WA USA; 4 University of Washington, Seattle, WA USA; 5 University of California Los Angeles, Los Angeles, CA USA; 6 Aarhus University Hospital, Aarhus, Denmark; 7 DANBIO Registry and Copenhagen Centre for Arthritis Research, Centre for Rheumatology and Spine Diseases VRR, Rigshospitalet, Glostrup, Denmark; 8 Department of Clinical Medicine, University of Copenhagen, Copenhagen, Denmark; 9 Department of Public Health, University of Copenhagen, Copenhagen, Denmark; 10 University of California San Francisco, San Francisco, CA USA

**Keywords:** Rheumatoid arthritis, Disease activity, Postpartum flare, Gene expression, RNA-seq

## Abstract

**Background:**

To evaluate our hypotheses that, when rheumatoid arthritis (RA) flares postpartum, gene expression patterns are altered compared to (a) healthy women, (b) RA women whose disease activity is low or in remission postpartum, and (c) pre-pregnancy expression profiles.

**Methods:**

Twelve women with RA and five healthy women were included in this pilot study. RA disease activity and postpartum flare were assessed using the Clinical Disease Activity Index (CDAI). Total RNA from frozen whole blood was used for RNA sequencing. Differential gene expression within the same women (within-group) over time, i.e., postpartum vs. third trimester (T3) or pre-pregnancy (T0), were examined, using a significance threshold of *q* < 0.05 and fold-change ≥ 2.

**Results:**

Nine of the women with RA experienced a flare postpartum (RA_Flare_), while three had low disease activity or were in remission (RA_NoFlare_) during that time frame. Numerous immune-related genes were differentially expressed postpartum (vs. T3) during a flare. Fold-changes in expression from T3 to postpartum were mostly comparable between the RA_Flare_ and healthy groups. At 3 months postpartum, compared to healthy women, several genes were significantly differentially expressed only among the RA_Flare_ women, and not among the RA_NoFlare_ women. Some of these genes were among those whose “normal” expression was significantly modulated postpartum, and the postpartum expression patterns were significantly altered during the RA flare. There were also some genes that were significantly differentially expressed in RA_Flare_ compared to both healthy and RA_NoFlare_ women, even though their expression was not significantly modulated postpartum. Furthermore, while postpartum expression profiles were similar to those at pre-pregnancy among healthy women, significant differences were found between those time points among the RA_Flare_ women.

**Conclusions:**

The large majority of gene expression changes between T3 and 3 months postpartum among RA women who flared postpartum reflected normal postpartum changes also seen among healthy women. Nonetheless, during a postpartum flare, a set of immune-related genes showed dysregulated expression compared to healthy women and women with RA whose disease activity was low or in remission during the same time frame, while other genes demonstrated significant differences in expression compared to RA pre-pregnancy levels.

**Supplementary Information:**

The online version contains supplementary material available at 10.1186/s13075-021-02418-w.

## Background

Rheumatoid arthritis (RA) is a chronic systemic inflammatory disease which can lead to progressive joint destruction and disability [1]. The disease, for which there is as yet no cure, affects approximately 1% of the adult world population, including 1.5 million adults in the USA [2]. Although pregnancy can induce a natural improvement of RA during pregnancy in 50–75% of women with the disease, this is followed by a predictable flare of the disease in the first 3 to 6 months after childbirth [3].

The biological basis of why the postpartum period predisposes women with RA to a flare has not been the subject of much research. While some studies have reported a decrease in anti-inflammatory cytokines [4], and an increase in T cell activation postpartum [5] in RA, it is not clear if these findings were associated with a flare. Gene expression studies reporting a persistence in monocyte activity and recurrence of lymphocyte gene activity [6], or a molecular activation of monocytes [7] postpartum, included mostly women who were in remission at the postpartum time point. Thus, altogether, the molecular mechanisms that lead to a flare postpartum in RA are still largely unknown. Of interest, systemic biological changes that occur postpartum in healthy women have also not been described.

In the present study, we have examined systemic gene expression changes from the 3rd trimester through to 6 months postpartum in a pilot subset of our prospective cohort of RA and healthy women [8], using RNA sequencing (RNA-seq). We evaluated our hypothesis that postpartum gene expression changes are altered when RA flares, compared to changes that occur among healthy women or women with RA who do not flare, within the same time frame. We examined temporal changes from the 3rd trimester to 3–6 months postpartum within each of these three groups of women to assess how biological changes that occur during a RA flare may overlap with and/or differ from what is observed among healthy women and RA women with low disease activity or in remission. Additionally, we examined whether the gene expression profile by 3 months postpartum reverts to the pre-pregnancy state in healthy women, and whether this is altered in RA.

## Subjects and methods

### Study subjects

Women with RA and healthy women of Danish descent were enrolled in our ongoing pregnancy study in Denmark, and prospectively followed until at least 6 months after childbirth. A set of 12 women with RA and 5 healthy women from our prospective cohort were included in the present pilot study. Women with RA fulfilled the 1987 revised American College of Rheumatology criteria for RA [9]. Study subjects with other autoimmune conditions were excluded so that those conditions do not confound the associations to be investigated. To enhance the chances of identifying genes that influence RA disease activity, only subjects of Danish ethnicity (parents and grandparents being ethnic Danes) were included. The study was approved by the Ethics Committee for Region Hovedstaden (Protocol #: H-2-2009-150) and the Data Protection Agency (Data processing ID: RH-2015-02; record #: i-suite 03601) in Denmark, and by the Children’s Hospital Oakland Research Institute Institutional Review Board in the USA (IRB #: 2009-073). All subjects provided written informed consent prior to enrollment.

### RA disease activity and postpartum flare

Clinical data, including data on disease activity measures, collected before pregnancy (T0), at the third trimester (T3), and at 3 and 6 months postpartum (PP3 and PP6, respectively) were used for analysis. The postpartum time point when disease activity was highest was denoted as PPmax. Women with missing data at T3, PP3, or PP6 were excluded. Because levels of acute phase reactants can fluctuate during and after pregnancy [10–12], RA disease activity was assessed at each time point using the Clinical Disease Activity Index (CDAI) [13], which does not include acute phase reactants. Women were classified as having a postpartum flare (RA_Flare_) if their CDAI state at T3 changed to a worse state by PP3 or PP6. Since this CDAI classification for RA flare is not yet validated, the presence of a flare was also assessed using a validated definition based on the disease activity score based on 28 joints (DAS28) [14]; DAS28 scores with 4 variables including C-reactive protein (DAS28CRP4) were computed [15] and an increase in DAS28 of 1.2 units or more between T3 and PPmax, or an increase of at least 0.6 units if the DAS28 at T3 was 3.2 or higher, was used to indicate a flare. Women with RA who did not flare but had low disease activity or were in remission postpartum were denoted as RA_NoFlare_.

### Sample processing

Total RNA was extracted from frozen whole blood using the PAXgene Blood RNA kit (Qiagen). RNA integrity assessment, cDNA library preparation, and RNA sequencing (RNA-seq) were performed as previously described [8].

### Bioinformatics analyses

The raw RNA-seq reads were pseudo-aligned to the reference human transcriptome (GRCh38; Ensembl release 98) and quantified using kallisto (version 0.42.4) [16]; transcripts that did not map to a primary chromosome (1–22, X, Y) or to the mitochondrial transcriptome (MT) were excluded from the reference. Biomart annotations were used to transform transcript-level counts to gene-level counts. Transcripts that mapped to pseudogenes, unannotated genes, and genes with low counts (less than 1 count per million in at least 25% of samples), as well as any remaining ribosomal RNA (rRNA) and globin transcripts, were filtered out. Any variation in sequencing depth was normalized using the Trimmed Mean of *M* values (TMM) algorithm as implemented in edgeR (version 3.26.8) [17].

### Statistical data analyses

Study subject characteristics and disease activity scores are presented as mean ± SD. Differential gene expression analysis was performed with edgeR (version 3.26.8) [18], using genewise generalized linear model (GLM) likelihood ratio tests. A negative binomial distribution was used to account for the over-dispersion in RNA-seq gene counts. To compare pairs of samples from the same women (within-group) at 2 time points, a paired sample design was used. The following within-group comparisons were performed: (1) PPmax vs. T3 (RA_Flare_ only), to identify postpartum gene expression changes when disease activity is maximal; (2) PP3 vs. T3, to identify postpartum expression changes in RA and healthy women; and (3) PP3 vs. T0, to determine whether, by 3 months postpartum, gene expression profiles had reverted to pre-pregnancy status. To identify genes that had significantly different expression levels postpartum in the RA groups compared to healthy women, between-group comparisons (RA_Flare_ or RA_NoFlare_ vs. healthy) were performed at both T3 and PP3. Since samples were sequenced in two batches, sequencing batch was included as a covariate in the GLMs to correct for any batch effects. Medications that the women with RA were taking were not adjusted for. However, to determine whether medications were a potential confounder, sensitivity analyses were performed for the PPmax vs. T3, PP3 vs. T3, and PP3 vs. T0 analyses within the RA_Flare_ group, adjusting for medications (yes/no—yes if the woman was currently on corticosteroids or disease-modifying anti-rheumatic drugs) as a time-varying covariate. A *q* value threshold of 0.05 was used to assess significance. Because sample sizes were small, fold-changes (FC) in expression were also used in the interpretation of results, focusing on genes differentially expressed by at least 2-fold change.

### Functional analysis

WebgestaltR [19] was used to perform enrichment analysis. Gene Ontology (GO) terms and Reactome pathways [20] were analyzed separately, and redundant terms were removed using Webgestalt’s “affinity propagation” function. Protein interactions were examined in Cytoscape [21], using data from the STRING database [22].

## Results

### Study subjects

Nine women with RA (RA_Flare_) and five healthy women had data and samples available at T3, PP3, and PP6. Another 3 women with RA (RA_NoFlare_) had data and samples available at T3 and PP3. Clinical characteristics and medication use among the 12 women with RA are shown in Table 1. RA duration was (mean ± SD) 4.8 ± 3.1 years for RA_Flare_ and 7.4 ± 4.4 years for RA_NoFlare_. The average age when they gave birth was 32.7 ± 4.8 years for the RA_Flare_ women, 30.7 ± 5.2 years for the RA_NoFlare_ women, and 31.9 ± 5.7 years for the healthy women. The average gestational age at birth was 282 ± 6 days for RA_Flare_, 283 ± 11 days for RA_NoFlare_, and 275 ± 9 days for healthy women.

**Table 1:** Clinical characteristics and medication use among the women with RA

Patient	RA duration* (years)	RF	ACPA	Medication use		
				3rd trimester (T3)	3 months postpartum (PP3)	6 months postpartum (PP6)
***RA***_***Flare***_						
1	10.3	+	+	PRDL + SSZ	PRDL	PRDL + corticosteroid (Diprospan)
2	4.7	+	+	PRDL + SSZ	–	PRDL + MTX
3	9.4	–	+	PRDL + SSZ	PRDL + SSZ	PRDL + glucocorticoid
4	2.6	NA	NA	–	PRDL + SSZ + HCQ	SSZ + HCQ + MTX
5	2.1	NA	+	PRDL	PRDL	PRDL
6	3.8	+	+	SSZ	PRDL + SSZ + MTX	SSZ + MTX
7	2.1	–	–	–	–	PRDL
8	5.4	NA	+	SSZ	PRDL + SSZ	SSZ
9	2.4	–	+	PRDL + SSZ	PRDL + SSZ	PRDL + MTX
***RA***_***NoFlare***_						
1	9.3	–	–	–	–	–
2	2.4	+	+	SSZ	MTX	MTX
3	10.5	–	–	Corticosteroid (Diprospan)	–	MTX + IFX

*RF* IgM rheumatoid factor, *ACPA* anti-citrullinated peptide antibodies, *HCQ* hydroxychloroquine, *MTX* methotrexate, *PRDL* prednisolone, *SSZ* sulfasalazine, *NA* not available

*RA duration at delivery

### RA disease activity and postpartum flare

**Fig. 1:**
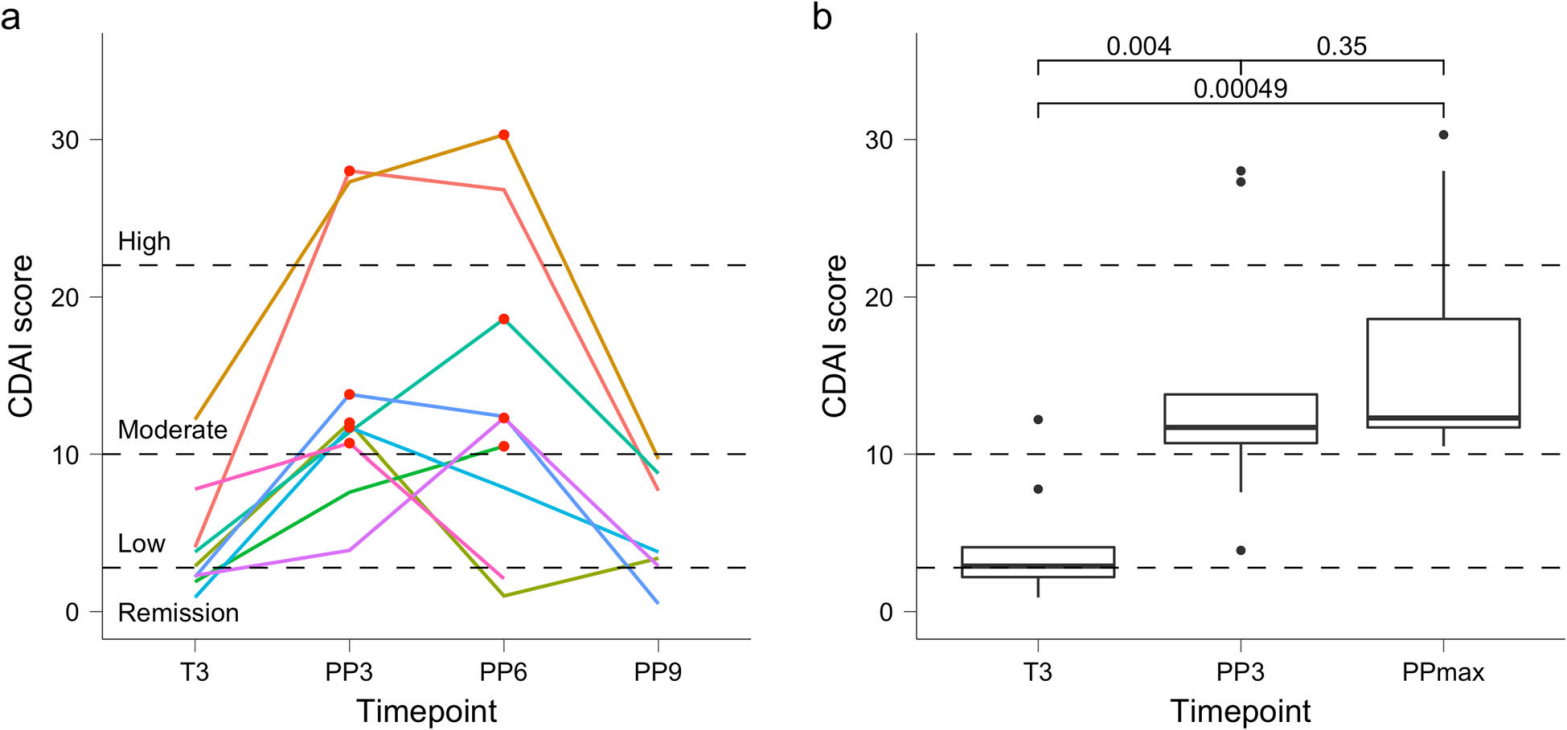
Changes in disease activity among women who flared postpartum. **a** Disease activity (CDAI) scores are shown at 3rd trimester (T3) and at 3, 6, and 9 months postpartum (PP3, PP6, and PP9, respectively) for the 9 women with RA who flared postpartum. The maximal postpartum disease activity recorded (PPmax) is marked by a red dot. **b** The mean CDAI scores increased significantly from T3 (mean ± SD, 4.2 ± 3.6) to both PP3 (14.0 ± 8.2, *p* = 0.004) and PPmax (16.4 ± 7.6, *p* = 0.0005)

At T3, the 9 RA_Flare_ women were in remission (*n* = 4) or had low (*n* = 4) or moderate (*n* = 1) disease activity (Fig. 1), with a CDAI score (mean ± SD) of 4.2 ± 3.6. Overall, there was a significant increase in CDAI scores from T3 to PP3 (14.0 ± 8.2, *p* = 0.004) or when disease activity was maximal (PPmax 16.4 ± 7.6, *p* = 0.0005). All 9 women had a flare postpartum, per CDAI classification; CDAI states changed from remission or low disease activity at T3 to moderate disease activity at PPmax for 7 women, and from low or moderate (T3) to high disease activity (PPmax) for 2 women. The CDAI and DAS28 scores were highly correlated (*r*^2^ = 0.88, *p* < 2E−16). Of the 9 RA_Flare_ women, 6 satisfied the DAS28 flare criteria. For the 3 RA_NoFlare_ women, disease activity remained low (*n* = 1) or in remission (*n* = 1) or improved from low to remission (*n* = 1) from T3 to PP3, with mean CDAI of 3.4 ± 0.7 (T3) and 2.0 ± 2.7 (PP3). Only 5 of the 9 women who flared postpartum had data available at pre-pregnancy (T0), and their CDAI score (mean ± SD) at that time point was 6.1 ± 3.4.

### Postpartum gene expression changes (vs. 3rd trimester) among RA and healthy women

#### RA_Flare_ women

Among the RA_Flare_ women, 272 genes were significantly differentially expressed (*q* < 0.05, fold-change (FC) ≥ 2) between T3 (remission/low disease activity) and PPmax (maximal disease activity: moderate/high), with most of those genes (*n* = 261, 96%) being under-expressed at PPmax (Supplementary Table S1). The under-expressed genes included many that were immune-related, such as alpha-defensin genes (DEFA1, DEFA1B, DEFA3, DEFA4), CD177, IFI27, IFIT1B, IL18R1, IL18RAP, IL1B, IL1R1, IL1R2, MMP8, MMP9, S100 genes (S100P, S100A8, S100A9, S100A12), TNFAIP6, MAOA, and CD274 (PD-L1). Genes that were over-expressed at PPmax (vs. T3) included the transcription factor GATA2, HDC, and mitochondrial genes MT-CO2, MT-ND3, and MT-ND6. Additional over-expressed genes that showed more modest fold-changes in expression included IL32, CD27, PTPRCAP, T cell receptor genes, and mitochondrial genes MT-CO1, MT-CO3 (1.5 ≤ FC < 2; *q* < 0.05). Expression levels of these genes did not change from pre-pregnancy to the 3rd trimester (data not shown). The differentially expressed genes with FC ≥ 2 (*n* = 272) were enriched in numerous GO terms related to immune function, including T helper 1 cell cytokine production (*q* = 5.6E−04) and innate immune response in mucosa (*q* = 7.7E−04), among others (Table 2). They were also enriched in immune-related Reactome pathways, such as neutrophil degranulation (*q* < 2.2E−16) and antimicrobial peptides (*q* = 1.9E−5). The protein products of a large proportion of these genes (*n* = 198, 73%) were found to interact with one another.

**Table 2:** The genes that were differentially expressed between the third trimester and the time point of maximal disease activity postpartum (PPmax) during the RA flare were enriched in these Gene Ontology (GO) terms

GO term	Description	Enrichment ratio	***q*** value
GO:0035744	T helper 1 cell cytokine production	34.7	5.6E−04
GO:0002227	Innate immune response in mucosa	19.3	7.7E−04
GO:0035456	Response to interferon-beta	10.5	9.1E−03
GO:0006778	Porphyrin-containing compound metabolic process	9.4	1.4E−02
GO:0031640	Killing of cells of other organism	9.3	4.5E−04
GO:0015701	Bicarbonate transport	8.3	2.2E−02
GO:0019730	Antimicrobial humoral response	8.2	2.5E−06
GO:0150076	Neuroinflammatory response	7.2	3.5E−02
GO:0030218	Erythrocyte differentiation	7.0	1.3E−04
GO:0002444	Myeloid leukocyte mediated immunity	7.0	< 2.2e−16
GO:0051851	Modification by host of symbiont morphology or physiology	6.9	6.5E−03
GO:0055076	Transition metal ion homeostasis	6.2	1.4E−04
GO:0097530	Granulocyte migration	6.2	1.5E−04
GO:0050729	Positive regulation of inflammatory response	5.3	2.8E−03
GO:1903900	Regulation of viral life cycle	5.1	3.2E−03
GO:0008645	Hexose transmembrane transport	4.6	4.3E−02
GO:0071222	Cellular response to lipopolysaccharide	4.3	3.1E−03
GO:0010950	Positive regulation of endopeptidase activity	4.3	1.1E−02
GO:0002700	Regulation of production of molecular mediator of immune response	4.1	4.4E−02
GO:0052548	Regulation of endopeptidase activity	3.2	2.2E−03

Only the top 20 GO terms (sorted by enrichment ratio) are shown

**Fig. 2:**
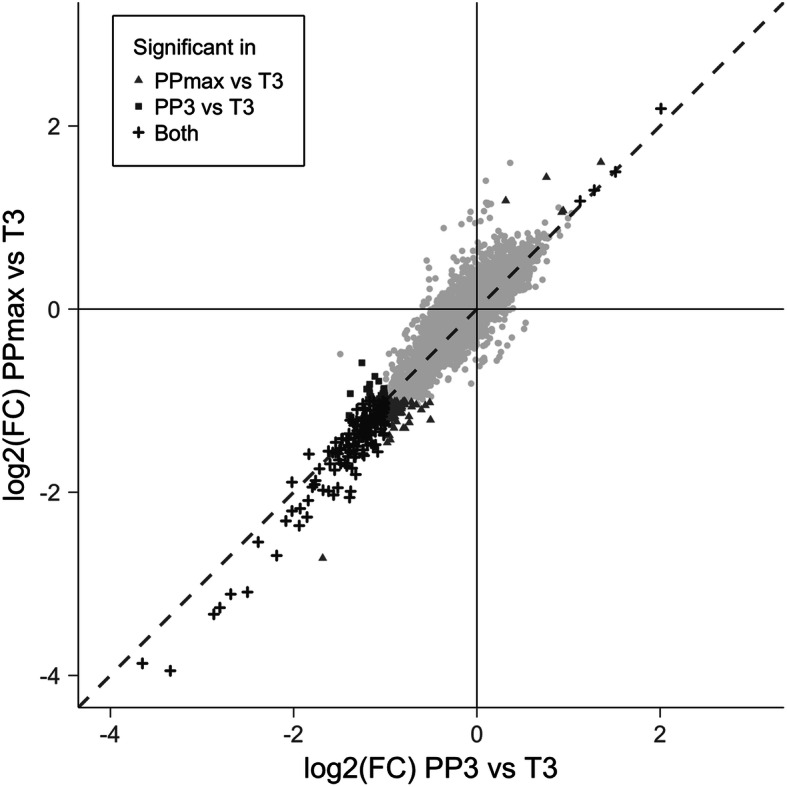
Postpartum changes in gene expression at PP3 or PPmax among women who flared. The changes in gene expression (log_2_-transformed fold-changes, FC) from the third trimester (T3) to 3 months postpartum (PP3), i.e., PP3 vs. T3 (x-axis), are plotted against those from T3 to the point of maximal disease activity (PPmax), i.e., PPmax vs. T3 (y-axis). All genes are shown, including those that were significantly differentially expressed in one or both comparisons and those that were not significantly differentially expressed in either analysis (gray dots)

When comparing the PP3 time point to T3 (instead of PPmax, which encompassed 2 time points: PP3 for 5 women and PP6 for 4 others), significant differential expression (*q* < 0.05, FC ≥ 2) was observed for 204 genes, most of which overlapped with those differentially expressed at PPmax (vs. T3) (Supplementary Table S1). Overall, the fold-changes in expression observed at PPmax and those at PP3 (both compared to T3) were highly correlated (pairwise correlation, 91.6%) (Fig. 2).

#### RA_NoFlare_ women

Of 51 genes that were significantly differentially expressed at PP3 (vs. T3), the majority (*n* = 38, i.e., 75%) were over-expressed postpartum, with fold-changes being higher (2.0 ≤ FC ≤ 4.6) than those observed among RA_Flare_ women; only one of these genes (NKX3-1) was also significantly over-expressed during a flare. There were 13 genes significantly under-expressed at PP3 (vs. T3) in the RA_NoFlare_ women, some of which overlapped with those under-expressed among RA_Flare_ women (OLFM4, MMP8, CAMP, CRISP3, ABCA13).

#### Healthy women

A total of 270 genes showed significant differential expression (*q* < 0.05, FC ≥ 2) between T3 and PP3, most (*n* = 254) being under-expressed at PP3 (Supplementary Table S2). Among the over-expressed genes (*n* = 16) were GATA2, S1PR5, and HDC. The differentially expressed genes were enriched in various GO terms, including several that were immune-related (Table 3), and in Reactome pathways such as neutrophil degranulation (*q* < 2.2E−16) and antimicrobial peptides (*q* = 1.3E−4).

**Table 3:** Genes that were differentially expressed between the third trimester (T3) and 3 months postpartum (PP3) among healthy women were enriched in these Gene Ontology (GO) terms

GO term	Description	Enrichment ratio	***q*** value
GO:0035744	T helper 1 cell cytokine production	25.7	1.5E−02
GO:0002227	Innate immune response in mucosa	19.0	8.2E−04
GO:0033008	Positive regulation of mast cell activation involved in immune response	15.8	4.9E−02
GO:0006779	Porphyrin-containing compound biosynthetic process	11.0	3.3E−02
GO:0015701	Bicarbonate transport	9.8	3.9E−03
GO:0031640	Killing of cells of other organism	9.1	4.6E−04
GO:0030218	Erythrocyte differentiation	8.8	2.8E−07
GO:0019730	Antimicrobial humoral response	8.7	3.1E−07
GO:1990266	Neutrophil migration	7.3	2.4E−05
GO:0002444	Myeloid leukocyte mediated immunity	6.6	< 2.2e−16
GO:0051851	Modification by host of symbiont morphology or physiology	5.9	3.9E−02
GO:0050729	Positive regulation of inflammatory response	5.7	6.6E−04
GO:2000379	Positive regulation of reactive oxygen species metabolic process	5.3	2.9E−02
GO:0055076	Transition metal ion homeostasis	5.1	3.7E−03
GO:0032680	Regulation of tumor necrosis factor production	4.2	4.5E−02
GO:0010951	Negative regulation of endopeptidase activity	4.1	1.3E−03
GO:0043281	Regulation of cysteine-type endopeptidase activity involved in apoptotic process	3.7	1.9E−02
GO:0002703	Regulation of leukocyte mediated immunity	3.5	4.2E−02
GO:0032496	Response to lipopolysaccharide	3.2	7.7E−03
GO:0034599	Cellular response to oxidative stress	3.2	2.0E−02

Only the top 20 GO terms (sorted by enrichment ratio) are shown

### Dysregulated postpartum gene expression during the RA flare

**Fig. 3:**
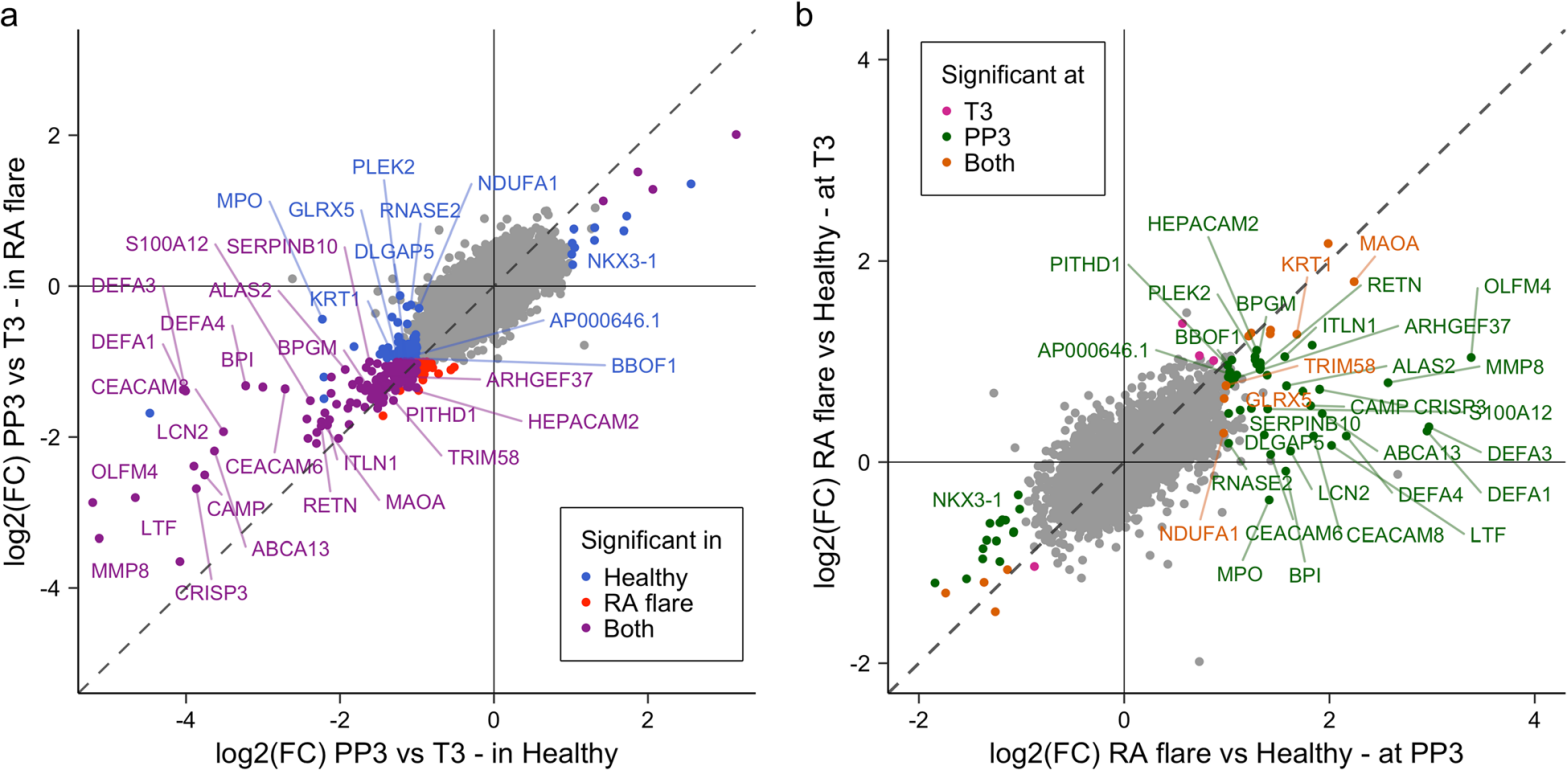
Some genes whose expression are modulated postpartum in healthy women demonstrate dysregulated expression during the RA postpartum flare. **a** Log_2_-transformed fold-changes in gene expression from the 3rd trimester (T3) to 3 months postpartum (PP3) are plotted for healthy women (*x*-axis) and for women with RA who flared postpartum (RA_Flare_) (*y*-axis). Several genes (*n* = 33) whose expression levels decreased significantly from T3 to PP3 among healthy women showed a significantly smaller decrease (or increase in the case of NKX3-1) in expression among the women with RA. **b** Log_2_-transformed fold-changes in gene expression for between-group (RA_Flare_ vs. healthy) analyses at PP3 (*x*-axis) are plotted against those at T3 (*y*-axis). The 33 genes that had a smaller decrease (or increase) in expression in RA_Flare_ postpartum (PP3 vs. T3 shown in **a**) did not have significantly different expression levels between the 2 groups of women at T3. At PP3, however, they had significantly (2–10-fold) higher (or lower) expression levels in RA_Flare_ compared to healthy women

The large majority of genes demonstrated similar expression changes from T3 to PP3, and in the same direction (increase or decrease) in both the RA_Flare_ and healthy groups. For a few genes, however, these temporal changes in expression were smaller in the RA_Flare_ than in the healthy group (Fig. 3a; Supplementary Table S2).

Genes were considered to have dysregulated expression during the postpartum flare if their expression at PP3 was significantly different in RA_Flare_—but not in the RA_NoFlare_—when compared to healthy women. There were a total of 68 such genes, with 50 over-expressed and 18 under-expressed in RA_Flare_ at PP3 (vs. healthy women). Of the 50 genes over-expressed specifically during the flare, a subset (*n* = 33) showed a significant decrease in expression postpartum (from T3 to PP3) among healthy women. These genes showed a smaller decrease in expression among the RA_Flare_ women such that by PP3, their levels remained higher than in healthy women (Fig. 3a; Supplementary Table S2). Between-group (RA_Flare_ vs. healthy) differential expression analyses revealed that, when RA flared at PP3, normalized expression levels for these 33 genes were *2–10-fold higher in RA* (*q* < 0.05) than in healthy women (Fig. 3b; Supplementary Table S2). Genes with the largest fold-changes in expression between the RA_Flare_ and healthy women (3.5 ≤ FC ≤ 10) were OLFM4, DEFA1, DEFA3, DEFA4, MMP8, MAOA, LTF, TUBB2A, ABCA13, CRISP3, CEACAM8, and CAMP. Interestingly, *when mean disease activity was low at T3*, expression levels of these genes (except MAOA and KRT1) were not significantly different between the RA_Flare_ and healthy women (Fig. 3b).

Among the RA_NoFlare_ women at PP3, most of the 68 genes with dysregulated expression during a flare were expressed at levels comparable to those in healthy women. Six of them (DEFA3, DEFA4, TUBB2A, RNF182, HLA-C, RNASE2) were found to be significantly over-expressed in RA_Flare_ (2 ≤ FC ≤ 7.8) compared to healthy as well as RA_NoFlare_ women (data not shown). Some were over-expressed (2 ≤ FC ≤ 3.6) among the RA_NoFlare_ women (vs. healthy) at PP3 (Supplementary Table S2), although these differences were not statistically significant. Of the 18 genes that were under-expressed during the RA flare (vs. healthy), 4 genes (IL5RA, SIGLEC8, CLEC4C, NKX3-1) were also significantly under-expressed during the flare compared to the RA_NoFlare_ group (data not shown). The 18 genes did not demonstrate any significant longitudinal changes in postpartum expression (T3 to PP3) in either RA or healthy women, except for the NKX3-1 gene which was over-expressed at PP3 (vs. T3) among healthy women.

**Fig. 4:**
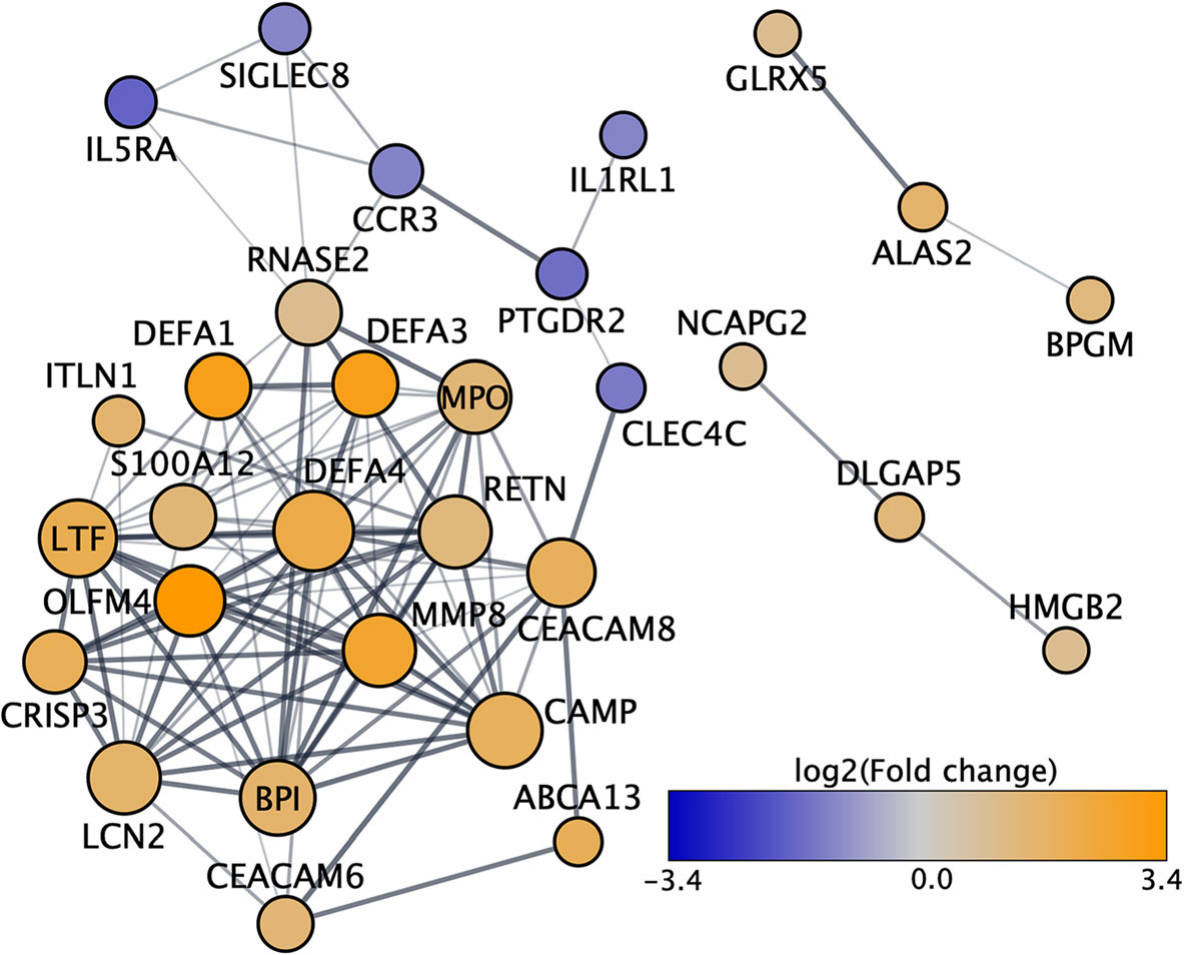
Interaction network of genes with dysregulated expression during the RA postpartum flare. The proteins encoded by a large proportion of the genes showing dysregulated expression during the RA postpartum flare belonged to a common interaction network

Functionally, the protein products encoded by several of the 68 genes that appeared to have dysregulated expression in RA during a flare were part of a common interaction network (Fig. 4). The 68 genes were enriched in GO terms such as neutrophil degranulation (*q* = 4.8E−12), defense response to bacterium (*q* = 1.5E−8), and disruption of cells of other organisms (*q* = 2.0E−5), and in Reactome gene sets relating to neutrophil degranulation (*q* = 8.2E−12) and antimicrobial peptides (*q* = 9.1E−6).

### Postpartum vs. pre-pregnancy expression profiles

Among healthy women, there was no significant differential expression at PP3 (*q* > 0.05), compared to pre-pregnancy (T0). In contrast, among the RA_Flare_ women (using only the women who had data available at T0; *n* = 5), 231 genes were significantly differentially expressed at PP3 compared to T0 (*q* < 0.05, FC ≥ 2). Of those, 159 were under-expressed at PP3 (e.g., CD177, PADI2, TLR9, NOTCH1, IL4R, IL12B, IL1R1). The 72 over-expressed genes included 7 T cell receptor alpha and beta variable genes, together with the TRAT1 (T Cell Receptor Associated Transmembrane Adaptor 1) gene, and 20 genes that encoded ribosomal proteins (RPL and RPS genes). Overall, the differentially expressed genes were enriched in the GO terms protein localization to endoplasmic reticulum (*q* < 2.2E−16), mRNA catabolic process (*q* = 9.8E−11), leukocyte degranulation (*q* = 2.6E−3), ribosome biogenesis (*q* = 7.7E−3), and negative regulation of immunoglobulin mediated immune response (*q* = 0.02)—and in the Reactome pathways eukaryotic translation elongation (*q* < 2.2E−16) and eukaryotic translation termination (*q* < 2.2E−16). Overall, these genes did not show significant differences in postpartum expression between the RA_Flare_ and healthy women, except for DLGAP5, NDUFA1, and RPL21.

### Sensitivity analysis

These results presented above for the (i) PPmax vs. T3, (ii) PP3 vs. T3, and (iii) PP3 vs. T0 comparisons within the RA_Flare_ group did not change when medications were included as a time-varying covariate in the models; the two sets of results were highly correlated (pairwise correlation ≥ 99.8%).

## Discussion

We present here the novel findings from our pilot study which aimed to evaluate two hypotheses. First, we hypothesized that gene expression changes accompanying the predictable postpartum flare in RA are altered compared to changes that occur among healthy women or women with RA whose disease activity is low or in remission within the same time frame. We further hypothesized that, during the RA flare, the postpartum gene expression profile does not revert back to the pre-pregnancy state.

In our dataset, postpartum changes in gene expression (from T3 to PP3) were mostly similar between RA_Flare_ and healthy women, suggesting that many of the changes observed during the RA flare are “normal.” However, while expression levels of most genes showing significant changes decreased postpartum in both groups of women, levels did not decrease to the same extent during a RA flare as they did in healthy women. They remained significantly higher in RA_Flare_ for numerous genes, including some whose increased expression have previously been associated with RA, such as DEFA1 and DEFA3 [23], LTF [24], MMP8 [25], CAMP [26], CEACAM8 [27], and CRISP3 [28]. We therefore speculate that postpartum expression of these genes may be dysregulated in RA women who flare, resulting in higher than normal postpartum levels which likely contribute to a pro-inflammatory environment, promoting a flare. This dysregulation appears to happen after childbirth; there were no significant differences in expression levels of these genes (except MAOA and KRT1) between RA and healthy women at the 3rd trimester, when the mean disease activity was low. Additionally, there were some genes that did not show longitudinal changes in expression postpartum, but their expression still appeared to be dysregulated postpartum during a flare, compared to both healthy and RA_NoFlare_ women at PP3. Among the genes under-expressed during the RA postpartum flare (vs. healthy or RA_NoFlare_ women at PP3) were the IL5RA and SIGLEC8 genes, both of which play a role in eosinophil apoptosis [29]. The mechanism by which these might trigger a flare is unclear. CLEC4C, which can dampen inflammation [30], was also under-expressed during the RA flare, compared to RA_NoFlare_ women. We found some candidate genes that were significantly over-expressed postpartum when RA flared (vs. healthy or RA_NoFlare_ women). Several of those have been implicated in pro-inflammatory processes; for example, HLA-C has been associated with RA [31]; RNF182 was previously reported to be over-expressed in RA [32], and TUBB2A was found to be over-expressed in psoriatic arthritis [33]. Given that the postpartum expression of these genes were dysregulated only during the postpartum flare, and that postpartum expression levels were comparable between healthy and RA_NoFlare_ women, we speculate that these genes may play a role in the postpartum flare of RA.

Our results show that, by 3 months postpartum, the gene expression profile among healthy women had reverted back to the pre-pregnancy state. In contrast, among the women with RA, some genes had significantly different expression levels at 3 months postpartum compared to before pregnancy. Contrary to what we expected, though, postpartum expression levels of those genes did not differ significantly between the RA and healthy women, suggesting that their postpartum expression in RA was not dysregulated. Furthermore, among the genes that were under-expressed postpartum (vs. pre-pregnancy) were PADI2 [34, 35], TLR9 [36], and NOTCH1 [37, 38], the increased expression of which have been implicated in RA. It is not clear why these pro-inflammatory genes are expressed at normal levels postpartum in RA, and why they are under-expressed compared to the pre-pregnancy state. We speculate that these genes are most likely not involved in the postpartum flare of the disease. We note that we had previously reported that type I interferon inducible genes may play a role in the pregnancy-induced improvement of RA [39]. Our data presented here suggest that the postpartum flare of RA is not simply a reversal of what happens during the pregnancy-induced improvement of RA, but that these could potentially represent two distinct biological phenomena that may or may not be the driven by the same underlying mechanism.

Although childbirth is often followed by a flare in RA, maternal systemic gene expression changes that accompany this flare had not previously been examined. Only one previous study had examined microarray-based gene expression at 6 months postpartum in RA [6, 7]. However, all but one of the 6 patients in that study were in remission (Rheumatoid Arthritis Disease Activity Index, RADAI ≤ 0.8) postpartum [6]. Thus, the gene expression changes reported did not reflect those that accompany a postpartum flare, and are not directly comparable to our findings. Most of the genes differentially expressed in RA were over-expressed at 6 months postpartum (vs. 3rd trimester), and did not overlap with those differentially expressed (*q* < 0.05, FC ≥ 2) in our data at either 3 or 6 months. In that study, postpartum (6 months) profiles of 8 healthy women were also examined, and a large proportion of the differentially expressed genes were under-expressed at 6 months postpartum (vs. 3rd trimester), as we also observed in our data; however, other than the DEFA1 and DEFA3 genes, there was little overlap with our findings among healthy women.

It is also not known whether the postpartum flare and a regular (not pregnancy-related) flare in RA share common underlying mechanisms or not. In a recent study of regular flares among 4 patients with RA [40], only few of the genes reported to have expression patterns associated with a flare overlapped with and showed expression changes in the same direction as the ones that we identified during the postpartum flare (HIC1, NRCAM, ALAS2, S100A12). The lack of commonality in the findings between this study and ours, even though Orange et al. used a less stringent significance threshold (*p* < 0.1) than we did, suggests that the mechanism(s) underlying the postpartum flare may indeed differ from those of a regular flare.

Our study has its strengths and limitations. The availability of longitudinal data from the same women enabled us to perform paired analyses, with each woman acting as her own control over time, thus controlling for unmeasured confounders. The use of RNA-seq technology to investigate gene expression was an advantage as it is more accurate and reliable than microarrays. Limitations included the following: First, our sample sizes were small. Nevertheless, the availability of longitudinal data from the same women before, during, and after pregnancy, and the ethnic homogeneity of the study population enhanced the statistical power and, to some extent, enabled us to overcome the limitations of a small sample size. Second, we did not examine changes in proportions of different cell types across time points because our goal was to identify overall systemic gene expression changes, resulting either from altered expression of specific genes or from differences in cell proportions. Third, there is a possibility that specific medications and/or dosage, which were not adjusted for in the analyses, may have confounded the results, although in our sensitivity analysis, we found no such indication.

We plan to use our larger cohort to follow up on these preliminary findings in specific cell types, and to examine epigenetic regulators of expression, including long non-coding RNAs, as potential drivers of the postpartum flare. It will also be important to further examine how the dysregulated gene expression pattern that we observed during the RA postpartum flare compares to those during regular RA flares in a larger set of patients with regular flares.

## Conclusions

Our findings from this pilot study suggest that, although postpartum gene expression patterns in RA were largely similar to those of healthy women, expression of some genes appeared to be dysregulated during a postpartum flare compared to both healthy women and RA women whose disease activity was low or in remission postpartum. We speculate that perhaps these genes could be involved in the postpartum flare of RA. Our results also show that by 3 months postpartum, expression profiles among healthy women reverted back to the pre-pregnancy state, whereas among RA women who flared postpartum, several genes involved in translation and immune processes among others did not revert to pre-pregnancy expression levels. These results are nevertheless preliminary and need to be replicated in a larger sample.

## Supplementary Information

**Additional file 1: Supplementary Table S1.** Genes differentially expressed between 3rd trimester and postpartum among 9 women with RA.

**Additional file 2: Supplementary Table S2.** Genes differentially expressed between 3rd trimester and 3 months postpartum in RA and/or healthy women.

## Data Availability

The data and materials are protected by the General Data Protection Regulation (GDPR) of the European Union (2016/679), and by the Danish Data Protection Act enacted in May 2018 to supplement the GDPR. The authors are legally forbidden from sharing data under the terms of their agreement with the Danish Data Protection Agency.

## Abbreviations

CDAI: Clinical Disease Activity Index
DAS28CRP4: Disease activity scores with 4 variables including C-reactive protein
FC: Fold-change
FDR: False discovery rate
GLM: Generalized linear model
GO: Gene Ontology
HCQ: Hydroxychloroquine
MTX: Methotrexate
PP3: 3 months postpartum
PP6: 6 months postpartum
PPmax: Postpartum time point when disease activity was highest
PRDL: Prednisolone
RA: Rheumatoid arthritis
RADAI: Rheumatoid Arthritis Disease Activity Index
RA_Flare_: Women with RA who flared postpartum
RA_NoFlare_: Women with RA whose disease activity was low or in remission postpartum
RNA-seq: RNA sequencing
rRNA: Ribosomal RNA
SD: Standard deviation
SSZ: Sulfasalazine
T0: Pre-pregnancy
T3: Third trimester
TMM: Trimmed Mean of *M* values
