# Supplementary material for: Is gene expression among women with rheumatoid arthritis dysregulated during a postpartum flare?
Source: Arthritis Res Ther. 2021 Jan 18;23:30. doi: 10.1186/s13075-021-02418-w (PMC7812735; doi:10.1186/s13075-021-02418-w)
Supplement: Supplementary file 1 — Additional file 1: Supplementary Table S1. Genes differentially expressed between 3rd trimester and postpartum among 9 women with RA. [file 13075_2021_2418_MOESM1_ESM.pdf]

Supplementary Table S1.

Genes differentially expressed between 3rd trimester and postpartum among 9 women with RA

Fold-changes (FCs) in expression at PPmax and at PP3 are shown (to 1 decimal place), compared to T3, together with q values, for genes that were significantly differentially expressed (FC≤-2 or FC≥2, q<0.05). FCs corresponding to a decrease in expression are shown as negative values. Gene expression changes that were statistically significant are shown in bold; those not statistically significant (q≥0.05) in either analysis are shown in red.

	PPmax vs T3		PP3 vs T3	
**Gene**	**Fold-change**	**q value**	**Fold-change**	**q value**
MMP8	**-15.4**	**4.5E-18**	**-10.2**	**1.3E-12**
CD177	**-14.6**	**2.2E-10**	**-12.6**	**2.5E-08**
OLFM4	**-10.1**	**2.3E-11**	**-7.3**	**2.5E-08**
LTF	**-9.6**	**4.7E-14**	**-7.0**	**7.2E-09**
CRISP3	**-8.6**	**2.1E-12**	**-6.4**	**3.6E-08**
CAMP	**-8.5**	**3.2E-13**	**-5.7**	**8.9E-08**
DEFA1B	**-6.6**	**4.0E-02**	-3.2	2.3E-01
ABCA13	**-6.5**	**2.5E-12**	**-4.5**	**3.4E-07**
LCN2	**-5.8**	**2.6E-11**	**-5.2**	**1.2E-08**
TCN1	**-5.2**	**5.9E-11**	**-3.8**	**2.6E-06**
ANXA3	**-5.0**	**4.1E-09**	**-4.2**	**8.3E-07**
RETN	**-4.8**	**1.1E-10**	**-3.6**	**4.8E-07**
GALNT14	**-4.6**	**2.1E-10**	**-4.0**	**9.3E-08**
CEACAM8	**-4.5**	**6.3E-09**	**-3.8**	**1.5E-06**
ORM1	**-4.3**	**1.3E-08**	**-3.6**	**4.9E-06**
DEFA3	**-4.2**	**1.1E-04**	**-2.6**	**2.0E-02**
ARG1	**-4.1**	**8.5E-07**	**-3.0**	**2.2E-03**
DEFA1	**-4.0**	**5.3E-05**	**-2.6**	**1.2E-02**
CHIT1	**-4.0**	**6.0E-11**	**-3.1**	**2.6E-06**
ITLN1	**-3.9**	**9.4E-07**	**-3.2**	**9.6E-05**
S100A12	**-3.9**	**1.1E-06**	**-2.9**	**5.6E-04**
MAOA	**-3.8**	**4.8E-07**	**-3.5**	**1.5E-05**
ITGB4	**-3.8**	**8.0E-12**	**-3.4**	**3.4E-09**
MCEMP1	**-3.7**	**4.8E-06**	**-4.1**	**1.0E-06**
CA1	**-3.7**	**1.9E-07**	**-3.4**	**2.8E-06**

DEFA4	**-3.5**	**1.9E-04**	**-2.5**	**1.7E-02**
OLR1	**-3.4**	**2.8E-07**	**-2.9**	**1.3E-05**
IFI27	**-3.3**	**2.2E-03**	**-3.3**	**2.5E-03**
CEACAM6	**-3.3**	**4.1E-05**	**-2.6**	**3.2E-03**
S100A8	**-3.3**	**3.1E-06**	**-2.7**	**4.5E-04**
CACNA1E	**-3.2**	**2.0E-07**	**-3.0**	**4.2E-06**
OSM	**-3.2**	**3.4E-10**	**-2.8**	**6.9E-07**
ATP2C2	**-3.2**	**1.3E-06**	**-2.7**	**1.3E-04**
IFIT1B	**-3.1**	**1.8E-07**	**-2.8**	**9.1E-06**
EPB42	**-3.1**	**1.1E-06**	**-2.5**	**3.1E-04**
BPI	**-3.1**	**5.8E-05**	**-2.5**	**3.1E-03**
SOCS3	**-3.1**	**9.3E-08**	**-2.7**	**8.5E-06**
KLF1	**-3.0**	**5.6E-07**	**-2.3**	**9.8E-04**
ALPL	**-3.0**	**3.8E-04**	**-3.6**	**1.8E-05**
PITHD1	**-3.0**	**6.4E-08**	**-2.5**	**4.7E-05**
CASP5	**-3.0**	**4.6E-05**	**-2.9**	**1.8E-04**
CD24	**-3.0**	**3.9E-06**	**-2.4**	**5.6E-04**
FCGR1A	**-3.0**	**2.8E-04**	**-2.6**	**3.7E-03**
MGAM2	**-3.0**	**8.4E-05**	**-3.0**	**1.6E-04**
KREMEN1	**-3.0**	**8.7E-06**	**-2.9**	**2.5E-05**
MS4A3	**-2.9**	**2.7E-04**	**-2.1**	**2.8E-02**
TRIM9	**-2.9**	**1.1E-05**	**-3.1**	**4.3E-06**
DSC2	**-2.9**	**1.4E-08**	**-2.6**	**5.0E-06**
ACSL1	**-2.9**	**8.6E-07**	**-2.4**	**9.9E-04**
XK	**-2.9**	**8.6E-07**	**-2.8**	**3.1E-06**
FCGR1B	**-2.9**	**7.3E-05**	**-2.8**	**2.6E-04**
CYSTM1	**-2.8**	**8.1E-10**	**-2.6**	**2.5E-07**
SPTB	**-2.8**	**2.8E-08**	**-2.5**	**4.3E-06**
SLPI	**-2.8**	**5.8E-05**	**-2.2**	**9.0E-03**
TLR5	**-2.8**	**2.1E-07**	**-2.6**	**5.0E-06**
ARHGEF37	**-2.8**	**5.6E-07**	**-2.3**	**1.8E-04**
PLSCR2	**-2.8**	**2.1E-08**	**-2.5**	**3.5E-06**
CA4	**-2.8**	**5.2E-06**	**-2.2**	**6.5E-03**
TMCC2	**-2.8**	**5.3E-07**	**-2.5**	**1.6E-05**
UBB	**-2.8**	**3.8E-04**	**-2.2**	**1.5E-02**
S100P	**-2.7**	**6.1E-05**	**-2.0**	**3.4E-02**
PFKFB3	**-2.7**	**4.7E-07**	**-2.9**	**1.2E-07**
ADM	**-2.7**	**3.4E-06**	**-2.4**	**1.6E-04**
BMX	**-2.7**	**1.1E-04**	-1.9	5.6E-02
FECH	**-2.7**	**1.4E-05**	**-2.7**	**3.1E-05**
UBE2O	**-2.7**	**4.2E-07**	**-2.8**	**9.3E-08**
BCL2A1	**-2.7**	**7.2E-06**	**-2.3**	**7.1E-04**
TENT5C	**-2.7**	**4.9E-05**	**-2.8**	**5.0E-05**

BPGM	**-2.7**	**3.0E-06**	**-2.5**	**2.5E-05**
DOK4	**-2.6**	**4.5E-09**	**-2.5**	**1.8E-07**
SELENBP1	**-2.6**	**1.0E-05**	**-2.4**	**1.5E-04**
FFAR3	**-2.6**	**6.0E-04**	**-2.4**	**3.4E-03**
OPLAH	**-2.6**	**2.3E-06**	-1.9	1.2E-02
CLEC4D	**-2.6**	**7.5E-07**	**-2.0**	**4.6E-03**
SIPA1L2	**-2.6**	**7.2E-06**	**-2.3**	**1.0E-03**
DOCK4	**-2.6**	**1.1E-06**	**-2.4**	**2.6E-05**
TRIM10	**-2.6**	**1.5E-06**	**-2.4**	**4.7E-05**
HEPACAM2	**-2.6**	**4.4E-05**	**-2.4**	**4.8E-04**
OSBP2	**-2.6**	**7.4E-05**	**-2.6**	**8.5E-05**
LRRN1	**-2.6**	**2.5E-07**	**-2.0**	**2.1E-03**
FOXO3	**-2.5**	**4.6E-08**	**-2.4**	**4.3E-06**
IL1R1	**-2.5**	**1.5E-04**	**-2.2**	**4.6E-03**
PGLYRP1	**-2.5**	**1.9E-02**	-2.3	5.1E-02
CR1	**-2.5**	**5.1E-07**	**-2.3**	**4.7E-05**
ALAS2	**-2.5**	**2.1E-04**	**-2.2**	**5.2E-03**
TRPM6	**-2.5**	**4.1E-09**	**-2.3**	**2.8E-06**
AIM2	**-2.5**	**2.9E-05**	**-2.5**	**9.9E-05**
TRIM58	**-2.5**	**7.2E-06**	**-2.4**	**4.4E-05**
SLC26A8	**-2.5**	**1.0E-05**	**-2.4**	**1.4E-04**
NLRC4	**-2.5**	**1.9E-08**	**-2.1**	**1.1E-04**
PFKFB2	**-2.5**	**4.4E-06**	-1.7	7.0E-02
MMP9	**-2.5**	**3.9E-03**	-1.7	1.7E-01
S100A9	**-2.5**	**3.9E-05**	-1.9	1.6E-02
PLIN4	**-2.4**	**7.8E-04**	**-2.3**	**5.6E-03**
FAM157B	**-2.4**	**3.8E-05**	**-2.3**	**3.6E-04**
SPTA1	**-2.4**	**3.9E-06**	**-2.3**	**4.6E-05**
NAIP	**-2.4**	**1.6E-08**	**-2.3**	**7.9E-07**
DYRK3	**-2.4**	**4.2E-07**	**-2.4**	**2.7E-06**
RHCE	**-2.4**	**2.5E-04**	**-2.4**	**4.6E-04**
KAZN	**-2.4**	**7.4E-07**	**-2.5**	**5.6E-07**
IL18RAP	**-2.4**	**1.1E-07**	-1.9	3.9E-03
GCA	**-2.4**	**3.9E-05**	**-2.0**	**5.6E-03**
PLSCR4	**-2.4**	**5.8E-05**	**-2.5**	**1.4E-05**
SLC14A1	**-2.4**	**4.0E-06**	**-2.4**	**2.2E-05**
DHRS13	**-2.4**	**3.2E-07**	**-2.3**	**7.9E-06**
F5	**-2.4**	**2.8E-07**	**-2.3**	**6.4E-06**
SLC22A4	**-2.4**	**5.1E-06**	**-2.0**	**1.9E-03**
SERPINB10	**-2.4**	**1.4E-04**	**-2.0**	**4.6E-03**
PGS1	**-2.4**	**1.9E-08**	**-2.2**	**1.9E-06**
MGAM	**-2.4**	**5.0E-04**	**-2.3**	**2.1E-03**
CBS	**-2.4**	**1.1E-03**	-1.7	1.4E-01

PLB1	-2.4	2.5E-07	-2.2	2.6E-05
ANK1	-2.4	3.9E-05	-2.5	2.8E-06
CDH19	-2.4	2.9E-04	-2.1	4.7E-03
RAP1GAP	-2.3	7.3E-03	-2.3	1.1E-02
TNFAIP6	-2.3	4.0E-04	-2.0	1.2E-02
ANKRD22	-2.3	6.8E-04	-1.8	5.5E-02
PROK2	-2.3	8.5E-05	-2.2	8.2E-04
PLSCR1	-2.3	1.1E-05	-2.1	4.4E-04
ALDH1A2	-2.3	1.8E-04	-2.1	2.2E-03
ACKR1	-2.3	9.4E-04	-2.5	1.8E-04
KCNJ15	-2.3	1.9E-05	-2.2	4.3E-04
BCAM	-2.3	7.5E-04	-2.6	7.0E-05
IL1R2	-2.3	5.1E-04	-1.4	3.6E-01
SLC4A1	-2.3	2.4E-04	-2.6	1.3E-05
HEPH	-2.3	5.0E-04	-2.0	1.4E-02
RGL4	-2.3	1.2E-08	-1.9	3.1E-04
KLHL2	-2.3	9.3E-07	-1.9	1.5E-03
CLEC5A	-2.3	5.6E-06	-1.9	3.1E-03
SEPTIN14	-2.3	2.6E-06	-2.4	2.0E-06
ST3GAL4	-2.3	1.5E-06	-2.3	1.5E-06
GYG1	-2.3	2.0E-08	-2.2	3.5E-07
STOM	-2.3	7.7E-10	-2.2	3.6E-08
SLFN14	-2.3	8.4E-06	-2.4	4.2E-06
LILRA5	-2.3	9.1E-06	-1.9	3.5E-03
GPX1	-2.3	1.4E-06	-1.9	4.9E-04
UGCG	-2.3	9.0E-08	-1.9	2.3E-04
ARHGEF12	-2.3	1.6E-06	-2.0	1.3E-04
CEBPE	-2.3	3.8E-04	-1.7	3.9E-02
LRG1	-2.3	4.1E-05	-2.0	2.4E-03
IL4R	-2.2	2.5E-07	-2.3	2.5E-07
ADRB3	-2.2	6.3E-02	-2.6	3.1E-02
GNG10	-2.2	1.1E-04	-1.9	5.2E-03
NCOA4	-2.2	5.1E-07	-2.1	2.5E-05
ITGA9	-2.2	1.5E-04	-1.9	5.8E-03
GRB10	-2.2	2.0E-03	-1.9	2.8E-02
MRC2	-2.2	5.6E-04	-2.1	1.9E-03
MED12L	-2.2	1.7E-06	-2.1	1.9E-05
MPP1	-2.2	1.9E-06	-2.0	7.2E-05
SLC37A3	-2.2	2.5E-07	-2.0	1.8E-04
KLC3	-2.2	1.3E-06	-1.8	3.9E-03
AC072022.2	-2.2	2.1E-03	-2.1	5.3E-03
GK	-2.2	3.5E-06	-2.1	7.9E-05
RIOK3	-2.2	1.4E-04	-2.1	8.9E-04

VNN1	**-2.2**	**2.0E-04**	-1.8	2.9E-02
BSG	**-2.2**	**1.1E-05**	**-2.0**	**2.9E-04**
WDFY3	**-2.2**	**1.6E-05**	**-2.0**	**1.1E-03**
NSUN7	**-2.2**	**9.4E-06**	-1.9	3.4E-03
BLVRB	**-2.2**	**8.1E-05**	-1.8	7.5E-03
PLEK2	**-2.2**	**5.8E-04**	-1.9	1.1E-02
WDR74	**-2.2**	**5.2E-03**	-1.9	3.4E-02
GADD45A	**-2.2**	**6.4E-06**	**-2.1**	**9.7E-05**
LY96	**-2.2**	**6.4E-06**	-1.9	8.3E-04
MCTP2	**-2.2**	**1.6E-06**	**-2.0**	**2.8E-04**
TTC26	**-2.2**	**6.0E-04**	**-2.2**	**3.8E-04**
SIAH2	**-2.1**	**7.5E-05**	**-2.1**	**2.7E-04**
TMOD1	**-2.1**	**5.1E-04**	**-2.3**	**9.2E-05**
KCNE1	**-2.1**	**2.5E-04**	-1.5	1.6E-01
PNP	**-2.1**	**1.3E-07**	-1.9	1.6E-05
ADGRG3	**-2.1**	**8.7E-03**	**-2.5**	**7.3E-04**
MRVI1	**-2.1**	**1.2E-04**	**-2.0**	**1.3E-03**
FCAR	**-2.1**	**3.7E-05**	-1.9	2.1E-03
FBXL13	**-2.1**	**1.6E-06**	-1.9	4.9E-04
PTX3	**-2.1**	**5.8E-03**	-1.8	5.9E-02
GYPB	**-2.1**	**2.0E-03**	**-2.0**	**3.8E-03**
AC011498.4	**-2.1**	**1.7E-04**	**-2.1**	**4.9E-04**
NIBAN1	**-2.1**	**3.3E-05**	-1.9	1.8E-03
CCNDBP1	**-2.1**	**3.8E-09**	-1.9	2.5E-05
CTSE	**-2.1**	**1.8E-03**	**-2.4**	**2.1E-04**
GMPR	**-2.1**	**3.1E-04**	-1.9	3.8E-03
TRIM6	**-2.1**	**8.1E-03**	-1.9	3.4E-02
TFDP1	**-2.1**	**4.6E-06**	**-2.1**	**2.6E-05**
MARCH8	**-2.1**	**1.1E-04**	**-2.0**	**6.3E-04**
BBOF1	**-2.1**	**1.8E-04**	-1.9	2.3E-03
NAMPT	**-2.1**	**2.1E-04**	-1.8	1.3E-02
PLAU	**-2.1**	**5.0E-04**	-1.8	1.9E-02
HIST3H2BB	**-2.1**	**7.2E-06**	-1.9	6.4E-04
TGM3	**-2.1**	**2.0E-03**	-1.6	1.0E-01
DNAJC25-GNG10	**-2.1**	**1.2E-04**	**-2.0**	**3.6E-04**
LMNB1	**-2.1**	**6.8E-06**	**-2.1**	**2.5E-05**
OR2W3	**-2.1**	**3.9E-04**	-1.9	7.0E-03
ARL4A	**-2.1**	**5.1E-04**	**-2.1**	**8.5E-04**
LGALS3	**-2.1**	**7.3E-05**	-1.9	7.2E-04
GABRR2	**-2.1**	**3.5E-05**	**-2.2**	**1.4E-05**
IL18R1	**-2.1**	**2.7E-05**	-1.5	1.7E-01
CREG1	**-2.1**	**3.9E-05**	-1.9	1.2E-03

TFR2	**-2.1**	**6.4E-06**	**-2.1**	**1.6E-05**
RHAG	**-2.1**	**8.0E-04**	**-2.0**	**1.6E-03**
PRDX2	**-2.1**	**7.5E-06**	**-2.0**	**2.5E-05**
LIPN	**-2.1**	**2.4E-04**	-1.8	1.3E-02
SLC1A3	**-2.1**	**1.3E-03**	-1.6	1.2E-01
TMTC1	**-2.1**	**5.3E-09**	-1.7	3.9E-04
SNCA	**-2.1**	**8.1E-04**	**-2.0**	**2.7E-03**
GLRX5	**-2.1**	**8.1E-05**	-1.9	1.2E-03
SLC2A3	**-2.1**	**1.1E-05**	-1.9	1.1E-03
ACSS3	**-2.1**	**2.5E-04**	-1.8	8.0E-03
VNN2	**-2.1**	**6.2E-04**	-1.7	3.5E-02
MKRN1	**-2.1**	**5.3E-05**	-1.8	2.4E-03
MAPK14	**-2.1**	**5.1E-06**	-1.8	2.8E-03
HMBS	**-2.1**	**6.5E-05**	**-2.1**	**7.7E-05**
MYL4	**-2.1**	**2.5E-02**	**-2.4**	**5.1E-03**
KIAA0825	**-2.0**	**3.5E-06**	-1.9	1.8E-04
E2F2	**-2.0**	**2.8E-04**	**-2.0**	**3.8E-04**
SLC1A5	**-2.0**	**2.6E-05**	**-2.0**	**6.2E-05**
SRPK1	**-2.0**	**4.4E-06**	**-2.0**	**5.9E-05**
MXI1	**-2.0**	**6.3E-04**	**-2.0**	**1.4E-03**
PLLP	**-2.0**	**1.5E-02**	-1.4	3.2E-01
B4GALT5	**-2.0**	**4.0E-04**	-1.9	2.6E-03
CYP26C1	**-2.0**	**1.1E-03**	-1.6	5.6E-02
BASP1	**-2.0**	**1.2E-03**	**-2.0**	**1.9E-03**
CDC34	**-2.0**	**1.6E-04**	-1.7	1.4E-02
SMIM5	**-2.0**	**4.4E-05**	-1.7	4.4E-03
PHTF1	**-2.0**	**2.0E-07**	-1.9	4.1E-05
PPP1R3B	**-2.0**	**1.1E-04**	-1.8	3.2E-03
C3orf86	**-2.0**	**2.3E-03**	-1.9	6.7E-03
RAB3IL1	**-2.0**	**1.4E-02**	**-2.0**	**1.5E-02**
IL1B	**-2.0**	**2.7E-04**	**-2.2**	**2.6E-05**
LYVE1	**-2.0**	**5.7E-03**	**-2.3**	**7.3E-04**
PYGL	**-2.0**	**1.7E-04**	-1.7	1.5E-02
HCAR3	**-2.0**	**1.1E-04**	**-2.0**	**2.9E-04**
DCAF12	**-2.0**	**1.1E-03**	-1.9	4.5E-03
HEMGN	**-2.0**	**4.0E-03**	**-2.2**	**8.6E-04**
ZNF442	**-2.0**	**6.0E-03**	-1.8	2.8E-02
SNX3	**-2.0**	**3.5E-05**	-1.9	1.6E-04
UBE2H	**-2.0**	**1.6E-07**	**-2.0**	**1.3E-06**
SLC25A37	**-2.0**	**7.1E-05**	-1.9	3.1E-04
GYPA	**-2.0**	**3.2E-03**	**-2.1**	**9.9E-04**
TGFA	**-2.0**	**5.8E-04**	-1.8	9.4E-03
DDX60L	**-2.0**	**2.6E-04**	-1.8	9.2E-03

FAM104A	**-2.0**	**5.9E-05**	-1.9	3.3E-04
MKNK1	**-2.0**	**9.7E-07**	-1.9	2.5E-05
AQP9	**-2.0**	**8.6E-04**	**-2.0**	**1.8E-03**
SERPINB1	**-2.0**	**2.0E-07**	-1.9	1.4E-05
TSTA3	**-2.0**	**5.4E-04**	**-2.1**	**2.5E-04**
MBOAT2	**-2.0**	**7.2E-06**	-1.9	4.6E-05
TGM2	**-2.0**	**7.4E-05**	**-2.3**	**3.5E-07**
FOLR3	**-2.0**	**7.9E-04**	-1.5	1.1E-01
CAPNS2	**-2.0**	**1.1E-04**	-1.9	7.8E-04
GCLC	**-2.0**	**4.0E-06**	**-2.0**	**3.0E-06**
ASPH	**-2.0**	**3.6E-04**	-1.7	3.1E-02
ABCG2	**-2.0**	**1.5E-03**	-1.9	4.9E-03
RP1	**-2.0**	**9.3E-04**	-1.7	1.8E-02
DYSF	**-2.0**	**9.5E-03**	**-2.1**	**5.1E-03**
GPR141	**-2.0**	**6.2E-05**	-1.7	9.0E-03
RNF14	**-2.0**	**3.8E-05**	-1.8	1.2E-03
DACH1	**-2.0**	**3.5E-04**	-1.8	3.3E-03
CD274	**-2.0**	**2.5E-02**	-1.9	4.5E-02
CASP4	**-2.0**	**6.5E-06**	-1.8	3.8E-04
KEL	**-2.0**	**1.5E-03**	**-2.0**	**6.4E-04**
CLTCL1	**-2.0**	**4.7E-06**	-1.6	1.5E-02
C4B	**-2.0**	**6.6E-03**	-1.9	1.2E-02
SLC8A1	**-2.0**	**3.0E-04**	-1.8	5.0E-03
TSPO2	-1.9	1.3E-02	**-2.0**	**8.3E-03**
TNS1	-1.9	9.2E-03	**-2.2**	**4.9E-04**
CREB5	-1.9	5.5E-04	**-2.0**	**6.6E-04**
MFSD2B	-1.9	1.1E-03	**-2.0**	**5.7E-04**
RNF10	-1.9	9.6E-05	**-2.0**	**7.7E-05**
LIMK2	-1.9	1.4E-03	**-2.1**	**3.0E-04**
HRH2	-1.9	1.4E-03	**-2.0**	**7.1E-04**
CISD2	-1.9	2.8E-03	**-2.0**	**2.2E-03**
FAM157A	-1.9	7.6E-02	**-2.6**	**2.0E-03**
OCLN	-1.9	4.8E-03	**-2.0**	**1.7E-03**
AQP1	-1.9	2.1E-03	**-2.0**	**5.4E-04**
CEACAM1	-1.9	2.6E-02	**-2.3**	**2.0E-03**
ANKRD40CL	-1.8	3.6E-02	**-2.0**	**1.5E-02**
CST7	-1.8	1.9E-02	**-2.3**	**3.6E-04**
JAZF1	-1.8	2.0E-04	**-2.0**	**2.8E-05**
USB1	-1.8	8.9E-04	**-2.0**	**7.7E-05**
SLC7A5	-1.8	3.5E-02	**-2.3**	**1.2E-03**
GABARAPL2	-1.8	4.1E-04	**-2.0**	**2.8E-05**
BCL6	-1.8	4.9E-02	**-2.2**	**1.3E-03**
SLC6A8	-1.7	7.7E-02	**-2.1**	**7.3E-03**

BCL2L1	-1.7	3.5E-02	**-2.0**	**3.0E-03**
LPO	-1.7	5.3E-02	**-2.2**	**8.3E-04**
NPB	-1.6	1.6E-01	**-2.0**	**3.9E-02**
NFIX	-1.5	3.1E-01	**-2.4**	**8.3E-03**
TIGD3	1.9	2.7E-03	**2.0**	**1.4E-03**
CRIP2	**2.0**	**5.4E-05**	**2.0**	**2.8E-04**
MT-CO2	**2.0**	**3.4E-02**	1.1	8.1E-01
SLC45A3	**2.1**	**2.2E-02**	1.9	6.2E-02
AL121845.2	**2.1**	**2.0E-02**	1.9	6.9E-02
PCSK5	**2.3**	**1.2E-02**	**2.2**	**2.0E-02**
MT-ND3	**2.3**	**5.5E-03**	1.2	5.5E-01
AKAP12	**2.5**	**7.0E-03**	**2.4**	**8.0E-03**
MT-ND6	**2.7**	**2.0E-02**	1.7	2.3E-01
FCER1A	**2.8**	**2.8E-04**	**2.9**	**4.6E-04**
GATA2	**3.0**	**1.8E-02**	2.6	5.7E-02
HDC	**4.6**	**1.3E-04**	**4.0**	**6.6E-04**
