# Supplementary material for: Is gene expression among women with rheumatoid arthritis dysregulated during a postpartum flare?
Source: Arthritis Res Ther. 2021 Jan 18;23:30. doi: 10.1186/s13075-021-02418-w (PMC7812735; doi:10.1186/s13075-021-02418-w)
Supplement: Supplementary file 2 — Additional file 2: Supplementary Table S2. Genes differentially expressed between 3rd trimester and 3 months postpartum in RA and/or healthy women. [file 13075_2021_2418_MOESM2_ESM.pdf]

**Supplementary Table S2.**

**Genes differentially expressed between 3rd trimester and 3 months postpartum in RA and/or healthy women**

Fold-changes (FCs) in expression (to 1 decimal place) and q values are shown for (a) the PP3 vs T3 analysis among RA women who flare postpartum ($RA_{Flare}$), (b) the PP3 vs T3 analysis in healthy women, and (c) the between-group (RA sub-groups vs healthy) comparisons performed at PP3. FCs corresponding to a decrease in expression are shown as negative values. Only genes that were significantly differentially expressed (FC≤-2 or FC≥2, q<0.05) in the PP3 vs T3 (in RA or healthy) or in $RA_{Flare}$ vs healthy at PP3 are shown. For each set of results, gene expression changes that were significant (FC≤-2 or FC≥2, q<0.05) are in bold, and changes that were not statistically significant (q≥0.05) are in red. For genes that demonstrated significant expression changes in $RA_{Flare}$, but not in $RA_{NoFlare}$ (vs healthy) at PP3, the FCs and q values are shown in blue.

| Gene | $RA_{Flare}$: PP3 vs T3 | | Healthy: PP3 vs T3 | | PP3: $RA_{Flare}$ vs Healthy | | PP3: $RA_{NoFlare}$ vs Healthy | |
|---|---|---|---|---|---|---|---|---|
| | **Fold-change** | **q value** | **Fold-change** | **q value** | **Fold-change** | **q value** | **Fold-change** | **q value** |
| OLFM4 | **-7.3** | **2.5E-08** | **-37.1** | **2.2E-15** | **10.4** | **3.4E-07** | 2.5 | 8.5E-01 |
| DEFA3 | **-2.6** | **2.0E-02** | **-16.1** | **1.5E-08** | **7.8** | **2.8E-05** | 1.3 | 1.0E+00 |
| DEFA1 | **-2.6** | **1.2E-02** | **-16.2** | **3.6E-10** | **7.7** | **4.4E-06** | 1.7 | 1.0E+00 |
| MMP8 | **-10.2** | **1.3E-12** | **-35.0** | **4.2E-17** | **5.9** | **3.7E-05** | 2.4 | 8.2E-01 |
| MAOA | **-3.5** | **1.5E-05** | **-4.7** | **1.1E-04** | **4.7** | **5.2E-05** | 3.6 | 1.2E-01 |
| DEFA4 | **-2.5** | **1.7E-02** | **-9.4** | **1.3E-06** | **4.5** | **1.8E-03** | -1.1 | 1.0E+00 |
| LTF | **-7.0** | **7.2E-09** | **-25.3** | **8.5E-15** | **4.1** | **1.8E-03** | 1.8 | 1.0E+00 |
| TUBB2A | -1.5 | 1.1E-01 | -1.4 | 4.8E-01 | **4.0** | **1.1E-06** | -1.7 | 1.0E+00 |
| ABCA13 | **-4.5** | **3.4E-07** | **-12.4** | **4.6E-12** | **3.8** | **6.6E-04** | 1.5 | 1.0E+00 |
| CRISP3 | **-6.4** | **3.6E-08** | **-14.6** | **4.5E-10** | **3.7** | **7.7E-03** | 2.1 | 1.0E+00 |
| CEACAM8 | **-3.8** | **1.5E-06** | **-11.4** | **8.4E-13** | **3.6** | **4.9E-04** | 2.0 | 9.4E-01 |
| CA1 | **-3.4** | **2.8E-06** | **-5.4** | **3.3E-07** | **3.6** | **1.4E-04** | **3.6** | **2.1E-02** |
| CAMP | **-5.7** | **8.9E-08** | **-13.5** | **4.5E-10** | **3.5** | **8.3E-03** | 2.2 | 9.4E-01 |
| PPEF2 | -1.1 | 7.7E-01 | -2.3 | 1.1E-01 | **3.3** | **1.0E-02** | 3.7 | 1.6E-01 |
| KRT1 | -1.9 | 2.1E-03 | **-2.5** | **4.7E-04** | **3.2** | **6.1E-07** | 2.4 | 5.5E-02 |

| | | | | | | | | |
|---|---|---|---|---|---|---|---|---|
| LCN2 | **-5.2** | **1.2E-08** | **-14.9** | **7.3E-14** | **3.1** | **6.7E-03** | 1.6 | 1.0E+00 |
| ALAS2 | **-2.2** | **5.2E-03** | **-3.8** | **9.3E-05** | **3.0** | **1.4E-03** | 1.9 | 8.8E-01 |
| BPI | **-2.5** | **3.1E-03** | **-8.0** | **4.4E-08** | **3.0** | **1.1E-02** | 1.0 | 1.0E+00 |
| ITLN1 | **-3.2** | **9.6E-05** | **-4.6** | **2.9E-04** | **3.0** | **1.6E-02** | 1.4 | 1.0E+00 |
| CEACAM6 | **-2.6** | **3.2E-03** | **-6.6** | **1.6E-06** | **2.7** | **3.4E-02** | 1.1 | 1.0E+00 |
| NPRL3 | -1.4 | 1.8E-01 | -1.5 | 2.2E-01 | **2.7** | **1.7E-05** | 1.6 | 9.9E-01 |
| PTGFR | -1.3 | 3.9E-01 | -1.4 | 4.6E-01 | **2.7** | **3.4E-03** | 1.3 | 1.0E+00 |
| MPO | -1.4 | 3.7E-01 | **-4.7** | **3.3E-05** | **2.7** | **1.4E-02** | -1.0 | 1.0E+00 |
| S100A12 | **-2.9** | **5.6E-04** | **-5.2** | **7.4E-05** | **2.6** | **3.8E-02** | 1.3 | 1.0E+00 |
| PLLP | -1.4 | 3.2E-01 | -2.1 | 1.3E-01 | **2.6** | **3.2E-02** | 2.6 | 5.2E-01 |
| DLGAP5 | -1.1 | 8.1E-01 | **-2.3** | **3.9E-02** | **2.6** | **1.6E-02** | 1.5 | 1.0E+00 |
| RETN | **-3.6** | **4.8E-07** | **-4.7** | **9.6E-06** | **2.5** | **2.4E-02** | 1.0 | 1.0E+00 |
| FAM83A | -1.5 | 2.4E-01 | -1.9 | 1.8E-01 | **2.5** | **3.5E-02** | 1.6 | 1.0E+00 |
| LGALS2 | 1.2 | 5.6E-01 | -1.1 | 8.0E-01 | **2.5** | **4.3E-04** | 1.3 | 1.0E+00 |
| SMIM1 | -1.3 | 3.6E-01 | -1.7 | 1.5E-01 | **2.5** | **2.0E-03** | **2.9** | **2.5E-02** |
| BPGM | **-2.5** | **2.5E-05** | **-3.1** | **9.4E-05** | **2.4** | **2.5E-03** | 2.3 | 1.8E-01 |
| HEPACAM2 | **-2.4** | **4.8E-04** | **-2.7** | **4.3E-03** | **2.4** | **1.3E-02** | 2.8 | 1.2E-01 |
| RNF182 | -1.2 | 4.9E-01 | -1.5 | 3.6E-01 | **2.4** | **8.3E-03** | -2.5 | 3.3E-01 |
| PLEK2 | -1.9 | 1.1E-02 | **-2.2** | **2.1E-02** | **2.4** | **7.2E-03** | 1.7 | 1.0E+00 |
| RGPD1 | 1.2 | 3.8E-01 | -1.3 | 4.2E-01 | **2.4** | **6.6E-04** | 1.2 | 1.0E+00 |
| IFIT1B | **-2.8** | **9.1E-06** | **-2.8** | **2.5E-03** | **2.4** | **1.3E-02** | **3.3** | **2.1E-02** |
| ADARB2 | -1.2 | 5.7E-01 | -1.1 | 7.9E-01 | **2.3** | **2.0E-03** | **2.5** | **4.2E-02** |
| SERPINB10 | **-2.0** | **4.6E-03** | **-3.1** | **3.7E-04** | **2.2** | **2.5E-02** | 1.3 | 1.0E+00 |
| BBOF1 | -1.9 | 2.3E-03 | **-2.3** | **5.4E-03** | **2.1** | **1.1E-02** | 1.8 | 8.4E-01 |
| ARHGEF37 | **-2.3** | **1.8E-04** | **-2.7** | **1.3E-03** | **2.1** | **2.9E-02** | 2.6 | 8.2E-02 |
| YOD1 | -1.8 | 8.0E-03 | -1.9 | 5.0E-02 | **2.1** | **1.6E-02** | 1.4 | 1.0E+00 |
| TRBV4-2 | 1.4 | 1.3E-01 | 1.2 | 7.0E-01 | **2.0** | **3.1E-03** | 1.1 | 1.0E+00 |
| AP000646.1 | -1.9 | 1.1E-02 | **-2.1** | **2.9E-02** | **2.0** | **4.9E-02** | 1.5 | 1.0E+00 |
| HLA-C | 1.0 | 8.4E-01 | -1.0 | 9.0E-01 | **2.0** | **2.1E-04** | -1.2 | 1.0E+00 |
| RPL21 | -1.2 | 5.7E-01 | -1.7 | 1.6E-01 | **2.0** | **4.9E-02** | 1.5 | 1.0E+00 |
| RNASE2 | -1.2 | 4.6E-01 | **-2.1** | **5.0E-03** | **2.0** | **8.4E-03** | -1.2 | 1.0E+00 |
| SMIM24 | -1.4 | 1.8E-01 | -1.6 | 2.1E-01 | **2.0** | **2.7E-02** | 1.4 | 1.0E+00 |
| PITHD1 | **-2.5** | **4.7E-05** | **-2.6** | **2.5E-03** | **2.0** | **4.2E-02** | 1.9 | 6.8E-01 |
| SLAMF8 | 1.0 | 9.7E-01 | -1.1 | 8.1E-01 | **2.0** | **2.2E-02** | -1.1 | 1.0E+00 |
| TRIM58 | **-2.4** | **4.4E-05** | **-2.8** | **2.5E-04** | **2.0** | **3.3E-02** | 1.9 | 6.4E-01 |
| HMGB2 | -1.1 | 7.7E-01 | -1.7 | 1.6E-01 | **2.0** | **4.5E-02** | 1.4 | 1.0E+00 |
| NCAPG2 | 1.2 | 1.3E-01 | 1.0 | 9.7E-01 | **2.0** | **6.0E-11** | 1.1 | 1.0E+00 |
| GLRX5 | -1.9 | 1.2E-03 | **-2.4** | **9.3E-04** | **2.0** | **1.5E-02** | 1.6 | 8.8E-01 |
| NDUFA1 | -1.2 | 4.5E-01 | **-2.0** | **4.8E-02** | **2.0** | **4.9E-02** | 1.2 | 1.0E+00 |
| REELD1 | -1.4 | 1.4E-01 | 1.1 | 8.4E-01 | **-2.0** | **4.9E-04** | -1.2 | 1.0E+00 |
| FAT4 | 1.2 | 4.5E-01 | 1.9 | 2.6E-03 | **-2.0** | **6.2E-05** | -1.4 | 1.0E+00 |
| TRBV28 | 1.2 | 4.6E-01 | 1.5 | 1.1E-01 | **-2.1** | **3.4E-04** | -1.6 | 8.6E-01 |
| FGFR2 | 1.3 | 2.5E-01 | 1.7 | 3.9E-02 | **-2.1** | **1.8E-04** | -1.4 | 1.0E+00 |

| | | | | | | | | |
|---|---|---|---|---|---|---|---|---|
| FADS2 | 1.2 | 2.2E-01 | 1.3 | 2.5E-01 | **-2.2** | **7.9E-09** | **-4.2** | **3.8E-14** |
| SIGLEC8 | -1.3 | 2.0E-01 | 1.1 | 6.7E-01 | **-2.2** | **2.2E-05** | -1.1 | 1.0E+00 |
| IL1RL1 | -1.1 | 7.5E-01 | 1.4 | 2.6E-01 | **-2.3** | **9.3E-05** | -1.2 | 1.0E+00 |
| CCR3 | 1.3 | 1.3E-01 | 1.6 | 6.4E-02 | **-2.3** | **1.1E-06** | -1.4 | 1.0E+00 |
| TRPC6 | -1.1 | 6.4E-01 | 1.4 | 3.4E-01 | **-2.3** | **1.1E-04** | -1.6 | 8.9E-01 |
| HIC1 | -1.4 | 2.3E-01 | -1.0 | 9.9E-01 | **-2.4** | **8.6E-04** | -1.5 | 1.0E+00 |
| NRCAM | 1.4 | 2.5E-01 | 1.2 | 6.8E-01 | **-2.4** | **2.5E-03** | -1.3 | 1.0E+00 |
| NKX3-1 | 1.5 | 4.4E-02 | **2.5** | **5.0E-05** | **-2.5** | **1.1E-06** | 1.4 | 1.0E+00 |
| SLC29A1 | -1.1 | 8.0E-01 | 1.4 | 4.6E-01 | **-2.5** | **1.2E-03** | -1.5 | 1.0E+00 |
| CLEC4C | -1.2 | 3.3E-01 | -1.1 | 7.9E-01 | **-2.6** | **1.1E-06** | 1.0 | 1.0E+00 |
| DDX11L10 | 1.0 | 9.7E-01 | 1.5 | 3.1E-01 | **-2.6** | **1.6E-04** | -1.7 | 1.0E+00 |
| SMPD3 | 1.0 | 9.5E-01 | 1.4 | 3.9E-01 | **-2.6** | **9.3E-05** | -1.2 | 1.0E+00 |
| PTGDR2 | 1.4 | 2.7E-01 | 1.9 | 1.4E-01 | **-2.9** | **8.3E-04** | -1.7 | 1.0E+00 |
| IL5RA | 1.3 | 4.9E-01 | 1.7 | 2.1E-01 | **-3.3** | **4.1E-05** | -1.1 | 1.0E+00 |
| ALOX15 | -1.2 | 7.4E-01 | 1.3 | 6.9E-01 | **-3.6** | **6.3E-03** | 1.0 | 1.0E+00 |
| DEFA1B | -3.2 | 2.3E-01 | **-22.2** | **3.5E-02** | 6.3 | 2.2E-01 | -3.1 | 1.0E+00 |
| S100A8 | **-2.7** | **4.5E-04** | **-4.3** | **9.6E-05** | 2.1 | 9.3E-02 | 1.3 | 1.0E+00 |
| CSTA | -1.2 | 5.3E-01 | **-2.2** | **3.3E-02** | 2.0 | 6.8E-02 | 1.3 | 1.0E+00 |
| FCGR1A | **-2.6** | **3.7E-03** | **-3.2** | **1.1E-02** | 2.0 | 2.2E-01 | 1.6 | 1.0E+00 |
| KLF1 | **-2.3** | **9.8E-04** | **-2.3** | **3.5E-02** | 2.0 | 9.3E-02 | 2.6 | 2.2E-01 |
| UBB | **-2.2** | **1.5E-02** | -2.2 | 1.1E-01 | 2.0 | 2.2E-01 | 1.8 | 1.0E+00 |
| MS4A3 | **-2.1** | **2.8E-02** | **-3.2** | **1.3E-02** | 2.0 | 2.3E-01 | 1.5 | 1.0E+00 |
| TENT5C | **-2.8** | **5.0E-05** | **-3.0** | **9.7E-04** | 1.9 | 8.8E-02 | 1.6 | 1.0E+00 |
| TMOD1 | **-2.3** | **9.2E-05** | **-2.7** | **5.2E-04** | 1.9 | 4.1E-02 | 1.6 | 1.0E+00 |
| NUDT4B | -1.8 | 3.1E-02 | **-2.7** | **2.3E-03** | 1.9 | 7.5E-02 | 1.3 | 1.0E+00 |
| TRIM10 | **-2.4** | **4.7E-05** | **-2.4** | **2.7E-03** | 1.9 | 4.2E-02 | **2.8** | **2.5E-02** |
| FAM210B | -1.9 | 4.3E-03 | **-2.3** | **3.7E-03** | 1.9 | 4.1E-02 | 1.7 | 9.8E-01 |
| RPL41 | -1.3 | 2.7E-01 | **-2.0** | **2.9E-02** | 1.9 | 4.0E-02 | 1.4 | 1.0E+00 |
| TSPO2 | **-2.0** | **8.3E-03** | **-2.9** | **3.7E-03** | 1.9 | 1.3E-01 | 2.6 | 2.2E-01 |
| ANLN | -1.4 | 2.0E-01 | **-2.2** | **2.3E-02** | 1.9 | 7.6E-02 | 1.1 | 1.0E+00 |
| ACKR1 | **-2.5** | **1.8E-04** | **-3.1** | **8.5E-04** | 1.9 | 1.3E-01 | 2.5 | 2.6E-01 |
| EPB42 | **-2.5** | **3.1E-04** | **-2.8** | **3.5E-03** | 1.9 | 1.3E-01 | 2.1 | 7.2E-01 |
| ARG1 | **-3.0** | **2.2E-03** | **-4.1** | **3.6E-03** | 1.9 | 3.4E-01 | 1.8 | 1.0E+00 |
| TCN1 | **-3.8** | **2.6E-06** | **-4.9** | **5.0E-05** | 1.9 | 2.3E-01 | 1.0 | 1.0E+00 |
| GYPB | **-2.0** | **3.8E-03** | **-2.1** | **3.6E-02** | 1.9 | 9.7E-02 | 2.0 | 6.2E-01 |
| SELENBP1 | **-2.4** | **1.5E-04** | **-3.4** | **4.9E-05** | 1.9 | 8.4E-02 | 2.0 | 5.6E-01 |
| SELENOK | -1.8 | 4.6E-04 | **-2.0** | **4.4E-03** | 1.9 | 1.3E-02 | 1.5 | 1.0E+00 |
| FECH | **-2.7** | **3.1E-05** | **-2.9** | **8.2E-04** | 1.9 | 9.7E-02 | 1.5 | 1.0E+00 |
| SPTB | **-2.5** | **4.3E-06** | **-3.6** | **3.3E-07** | 1.9 | 4.5E-02 | 1.7 | 8.6E-01 |
| KLC3 | -1.8 | 3.9E-03 | **-2.0** | **8.9E-03** | 1.9 | 3.5E-02 | 1.8 | 7.4E-01 |
| SIAH2 | **-2.1** | **2.7E-04** | **-2.5** | **7.2E-04** | 1.9 | 4.1E-02 | 1.6 | 9.9E-01 |
| S100A9 | -1.9 | 1.6E-02 | **-2.8** | **2.7E-03** | 1.8 | 1.2E-01 | 1.2 | 1.0E+00 |
| GMPR | -1.9 | 3.8E-03 | **-2.4** | **3.9E-03** | 1.8 | 7.4E-02 | 1.8 | 8.6E-01 |

| | | | | | | | | |
|---|---|---|---|---|---|---|---|---|
| TNFAIP6 | -2.0 | 1.2E-02 | -2.0 | 8.2E-02 | 1.8 | 1.6E-01 | 1.3 | 1.0E+00 |
| RHAG | -2.0 | 1.6E-03 | -2.1 | 1.9E-02 | 1.8 | 7.4E-02 | 2.0 | 5.2E-01 |
| TMCC2 | -2.5 | 1.6E-05 | -2.6 | 1.2E-03 | 1.8 | 7.4E-02 | 1.8 | 7.7E-01 |
| MMP9 | -1.7 | 1.7E-01 | -3.5 | 9.5E-03 | 1.8 | 3.5E-01 | 1.2 | 1.0E+00 |
| MXI1 | -2.0 | 1.4E-03 | -2.2 | 6.8E-03 | 1.8 | 6.5E-02 | 1.6 | 1.0E+00 |
| ELOB | -1.6 | 9.5E-03 | -2.1 | 2.7E-03 | 1.8 | 1.6E-02 | 1.5 | 1.0E+00 |
| DNAJC6 | -1.8 | 3.4E-02 | -2.2 | 2.6E-02 | 1.8 | 1.4E-01 | 1.7 | 1.0E+00 |
| MKRN1 | -1.8 | 2.4E-03 | -2.2 | 2.7E-03 | 1.8 | 4.2E-02 | 1.6 | 8.6E-01 |
| SNCA | -2.0 | 2.7E-03 | -2.0 | 3.0E-02 | 1.8 | 8.9E-02 | 1.4 | 1.0E+00 |
| RIOK3 | -2.1 | 8.9E-04 | -2.0 | 2.6E-02 | 1.8 | 8.8E-02 | 1.5 | 1.0E+00 |
| SLC14A1 | -2.4 | 2.2E-05 | -2.1 | 6.8E-03 | 1.8 | 6.4E-02 | 2.2 | 1.8E-01 |
| RBM38 | -1.8 | 4.3E-03 | -2.2 | 3.7E-03 | 1.8 | 5.6E-02 | 1.5 | 1.0E+00 |
| ANKRD9 | -1.7 | 1.3E-02 | -2.3 | 1.2E-03 | 1.8 | 4.9E-02 | 1.3 | 1.0E+00 |
| SOX6 | -1.9 | 3.4E-03 | -2.2 | 5.0E-03 | 1.8 | 7.1E-02 | 1.7 | 9.4E-01 |
| CLEC4D | -2.0 | 4.6E-03 | -2.3 | 1.4E-02 | 1.8 | 1.3E-01 | 1.1 | 1.0E+00 |
| OR2W3 | -1.9 | 7.0E-03 | -2.7 | 7.5E-04 | 1.8 | 9.3E-02 | 1.1 | 1.0E+00 |
| ANXA3 | -4.2 | 8.3E-07 | -4.9 | 9.0E-05 | 1.8 | 3.3E-01 | 2.2 | 8.6E-01 |
| GYPA | -2.1 | 9.9E-04 | -2.1 | 3.3E-02 | 1.8 | 1.3E-01 | 1.9 | 7.4E-01 |
| FCER1G | -1.8 | 7.2E-04 | -2.6 | 8.0E-06 | 1.8 | 2.1E-02 | 1.3 | 1.0E+00 |
| SPTA1 | -2.3 | 4.6E-05 | -2.2 | 5.5E-03 | 1.7 | 9.3E-02 | 2.3 | 1.7E-01 |
| SLC25A37 | -1.9 | 3.1E-04 | -2.3 | 4.7E-04 | 1.7 | 4.4E-02 | 1.6 | 8.6E-01 |
| BLVRB | -1.8 | 7.5E-03 | -2.1 | 1.8E-02 | 1.7 | 1.1E-01 | 1.6 | 1.0E+00 |
| ARHGEF12 | -2.0 | 1.3E-04 | -2.0 | 7.4E-03 | 1.7 | 5.1E-02 | 1.6 | 8.8E-01 |
| HIST3H2BB | -1.9 | 6.4E-04 | -2.2 | 1.1E-03 | 1.7 | 4.2E-02 | 1.4 | 1.0E+00 |
| CD177 | -12.6 | 2.5E-08 | -16.9 | 3.9E-06 | 1.7 | 5.9E-01 | 1.6 | 1.0E+00 |
| DCAF12 | -1.9 | 4.5E-03 | -2.2 | 7.8E-03 | 1.7 | 1.2E-01 | 1.6 | 1.0E+00 |
| HEMGN | -2.2 | 8.6E-04 | -2.3 | 1.5E-02 | 1.7 | 1.7E-01 | 1.7 | 1.0E+00 |
| MARCH8 | -2.0 | 6.3E-04 | -2.2 | 4.4E-03 | 1.7 | 9.6E-02 | 1.7 | 7.9E-01 |
| MCEMP1 | -4.1 | 1.0E-06 | -5.3 | 3.6E-05 | 1.7 | 3.7E-01 | 1.2 | 1.0E+00 |
| SLC4A1 | -2.6 | 1.3E-05 | -3.3 | 1.6E-05 | 1.7 | 1.4E-01 | 1.9 | 6.2E-01 |
| CDC34 | -1.7 | 1.4E-02 | -2.0 | 2.4E-02 | 1.7 | 1.2E-01 | 1.5 | 1.0E+00 |
| HAGH | -1.7 | 3.1E-03 | -2.1 | 3.6E-03 | 1.7 | 6.8E-02 | 1.5 | 1.0E+00 |
| IFI27 | -3.3 | 2.5E-03 | -2.7 | 8.8E-02 | 1.7 | 5.3E-01 | 1.1 | 1.0E+00 |
| SLPI | -2.2 | 9.0E-03 | -3.0 | 5.2E-03 | 1.7 | 3.5E-01 | 1.2 | 1.0E+00 |
| SLC1A5 | -2.0 | 6.2E-05 | -2.2 | 7.0E-04 | 1.7 | 5.5E-02 | 1.7 | 7.3E-01 |
| TFDP1 | -2.1 | 2.6E-05 | -2.2 | 4.7E-04 | 1.7 | 5.5E-02 | 1.3 | 1.0E+00 |
| CLTCL1 | -1.6 | 1.5E-02 | -2.0 | 2.3E-03 | 1.7 | 4.9E-02 | -1.1 | 1.0E+00 |
| DYRK3 | -2.4 | 2.7E-06 | -2.3 | 9.7E-04 | 1.7 | 9.6E-02 | 2.1 | 1.8E-01 |
| AIF1 | -1.4 | 1.7E-01 | -2.4 | 2.7E-03 | 1.6 | 1.4E-01 | 1.2 | 1.0E+00 |
| CTSE | -2.4 | 2.1E-04 | -2.1 | 2.9E-02 | 1.6 | 2.2E-01 | 1.5 | 1.0E+00 |
| BCL2L1 | -2.0 | 3.0E-03 | -2.3 | 4.3E-03 | 1.6 | 1.7E-01 | 1.5 | 1.0E+00 |
| MPP1 | -2.0 | 7.2E-05 | -2.0 | 4.4E-03 | 1.6 | 8.5E-02 | 1.5 | 1.0E+00 |
| MYL4 | -2.4 | 5.1E-03 | -2.8 | 1.0E-02 | 1.6 | 3.8E-01 | 2.0 | 9.8E-01 |

| | | | | | | | | |
|---|---|---|---|---|---|---|---|---|
| FFAR3 | **-2.4** | **3.4E-03** | **-2.9** | **9.2E-03** | 1.6 | 3.9E-01 | 1.3 | 1.0E+00 |
| RNF10 | **-2.0** | **7.7E-05** | **-2.4** | **9.4E-05** | 1.6 | 6.4E-02 | 1.5 | 9.4E-01 |
| TMEM63B | -1.8 | 1.2E-03 | **-2.4** | **2.3E-04** | 1.6 | 8.0E-02 | 1.3 | 1.0E+00 |
| E2F2 | **-2.0** | **3.8E-04** | **-2.2** | **3.7E-03** | 1.6 | 1.3E-01 | 1.5 | 1.0E+00 |
| SLC25A39 | -1.6 | 7.9E-02 | **-2.0** | **3.9E-02** | 1.6 | 2.0E-01 | 1.4 | 1.0E+00 |
| CISD2 | **-2.0** | **2.2E-03** | **-2.1** | **1.3E-02** | 1.6 | 1.8E-01 | 1.7 | 9.1E-01 |
| PRDX2 | **-2.0** | **2.5E-05** | **-2.4** | **8.4E-05** | 1.6 | 7.9E-02 | 1.7 | 6.1E-01 |
| ABCG2 | -1.9 | 4.9E-03 | **-2.0** | **2.5E-02** | 1.6 | 2.2E-01 | 2.3 | 1.8E-01 |
| LGALS3 | -1.9 | 7.2E-04 | **-2.4** | **8.5E-04** | 1.6 | 1.3E-01 | 1.5 | 1.0E+00 |
| XK | **-2.8** | **3.1E-06** | **-3.0** | **2.4E-04** | 1.6 | 2.5E-01 | 2.0 | 5.6E-01 |
| SNX3 | -1.9 | 1.6E-04 | **-2.1** | **2.7E-03** | 1.6 | 9.6E-02 | 1.4 | 1.0E+00 |
| RAP1GAP | **-2.3** | **1.1E-02** | **-4.3** | **5.7E-04** | 1.6 | 5.0E-01 | 2.1 | 9.5E-01 |
| GPX1 | -1.9 | 4.9E-04 | **-2.0** | **7.5E-03** | 1.6 | 1.4E-01 | 1.3 | 1.0E+00 |
| FAM104A | -1.9 | 3.3E-04 | **-2.0** | **6.2E-03** | 1.6 | 1.3E-01 | 1.3 | 1.0E+00 |
| SLC6A9 | -1.9 | 1.3E-02 | **-2.1** | **3.5E-02** | 1.5 | 3.2E-01 | 1.9 | 8.0E-01 |
| TXN | -1.7 | 1.5E-02 | **-2.1** | **1.3E-02** | 1.5 | 2.5E-01 | 1.3 | 1.0E+00 |
| ANK1 | **-2.5** | **2.8E-06** | **-2.9** | **4.3E-05** | 1.5 | 2.2E-01 | 1.7 | 9.1E-01 |
| ORM1 | **-3.6** | **4.9E-06** | **-4.5** | **9.2E-05** | 1.5 | 4.9E-01 | 1.7 | 1.0E+00 |
| HEBP1 | -1.7 | 1.0E-02 | **-2.0** | **8.1E-03** | 1.5 | 1.8E-01 | 1.7 | 8.0E-01 |
| FOXO3 | **-2.4** | **4.3E-06** | **-2.3** | **1.3E-03** | 1.5 | 2.0E-01 | 1.5 | 1.0E+00 |
| BMX | -1.9 | 5.6E-02 | **-2.4** | **4.9E-02** | 1.5 | 5.3E-01 | 1.2 | 1.0E+00 |
| PGLYRP1 | -2.3 | 5.1E-02 | **-4.6** | **3.6E-03** | 1.5 | 6.3E-01 | 1.2 | 1.0E+00 |
| VNN1 | -1.8 | 2.9E-02 | **-2.3** | **1.9E-02** | 1.5 | 3.9E-01 | 1.0 | 1.0E+00 |
| UBE2O | **-2.8** | **9.3E-08** | **-2.7** | **1.0E-04** | 1.5 | 2.5E-01 | 2.0 | 3.1E-01 |
| ARL4A | **-2.1** | **8.5E-04** | -1.9 | 6.4E-02 | 1.5 | 3.5E-01 | 1.4 | 1.0E+00 |
| DPM2 | -1.6 | 1.8E-02 | **-2.0** | **7.2E-03** | 1.5 | 2.6E-01 | 1.5 | 1.0E+00 |
| NCOA4 | **-2.1** | **2.5E-05** | -1.9 | 1.1E-02 | 1.5 | 2.2E-01 | 1.5 | 1.0E+00 |
| TSTA3 | **-2.1** | **2.5E-04** | **-2.0** | **2.1E-02** | 1.5 | 3.2E-01 | 1.3 | 1.0E+00 |
| BSG | **-2.0** | **2.9E-04** | **-2.1** | **5.0E-03** | 1.4 | 3.1E-01 | 1.4 | 1.0E+00 |
| MFSD2B | **-2.0** | **5.7E-04** | **-2.2** | **3.6E-03** | 1.4 | 3.4E-01 | 1.5 | 1.0E+00 |
| ST6GALNAC4 | -1.7 | 2.9E-02 | **-2.4** | **2.7E-03** | 1.4 | 3.7E-01 | 1.3 | 1.0E+00 |
| CHIT1 | **-3.1** | **2.6E-06** | **-3.7** | **4.3E-05** | 1.4 | 4.9E-01 | 1.1 | 1.0E+00 |
| GABARAPL2 | **-2.0** | **2.8E-05** | -1.9 | 5.2E-03 | 1.4 | 2.2E-01 | 1.3 | 1.0E+00 |
| NFIX | **-2.4** | **8.3E-03** | **-2.9** | **1.4E-02** | 1.4 | 6.0E-01 | 1.8 | 1.0E+00 |
| KEL | **-2.0** | **6.4E-04** | **-2.4** | **1.8E-03** | 1.4 | 3.9E-01 | 1.3 | 1.0E+00 |
| STOM | **-2.2** | **3.6E-08** | **-2.0** | **5.4E-04** | 1.4 | 1.5E-01 | 1.1 | 1.0E+00 |
| TDRD9 | -1.7 | 6.6E-03 | **-2.0** | **5.7E-03** | 1.4 | 2.8E-01 | -1.0 | 1.0E+00 |
| AFF2 | -1.6 | 2.4E-02 | **-2.4** | **8.2E-04** | 1.4 | 3.4E-01 | 1.4 | 1.0E+00 |
| OLR1 | **-2.9** | **1.3E-05** | **-3.4** | **2.7E-04** | 1.4 | 5.3E-01 | 1.2 | 1.0E+00 |
| TECTA | -1.5 | 1.3E-02 | **-2.0** | **2.3E-03** | 1.4 | 2.5E-01 | 1.2 | 1.0E+00 |
| AC104389.6 | -2.8 | 5.8E-02 | **-4.6** | **2.1E-02** | 1.4 | 7.7E-01 | 1.5 | 1.0E+00 |
| SLC26A8 | **-2.4** | **1.4E-04** | **-2.7** | **1.3E-03** | 1.4 | 4.8E-01 | 1.3 | 1.0E+00 |
| AIM2 | **-2.5** | **9.9E-05** | **-2.5** | **4.4E-03** | 1.4 | 5.1E-01 | 1.1 | 1.0E+00 |

| | | | | | | | | |
|---|---|---|---|---|---|---|---|---|
| NDUFB3 | -1.7 | 1.4E-02 | **-2.0** | **1.9E-02** | 1.4 | 4.0E-01 | 1.5 | 1.0E+00 |
| PLSCR4 | **-2.5** | **1.4E-05** | **-2.2** | **1.8E-02** | 1.4 | 5.0E-01 | 1.5 | 1.0E+00 |
| CACNA1E | **-3.0** | **4.2E-06** | **-3.2** | **5.2E-04** | 1.4 | 5.6E-01 | 1.3 | 1.0E+00 |
| FCAR | -1.9 | 2.1E-03 | **-2.2** | **5.6E-03** | 1.4 | 4.5E-01 | 1.2 | 1.0E+00 |
| AQP1 | **-2.0** | **5.4E-04** | **-2.4** | **1.3E-03** | 1.4 | 4.6E-01 | 1.4 | 1.0E+00 |
| DMTN | -1.7 | 7.9E-02 | **-2.3** | **4.0E-02** | 1.4 | 5.9E-01 | 1.3 | 1.0E+00 |
| FCGR1B | **-2.8** | **2.6E-04** | **-2.9** | **5.7E-03** | 1.4 | 6.3E-01 | 1.3 | 1.0E+00 |
| GADD45A | **-2.1** | **9.7E-05** | **-2.1** | **6.2E-03** | 1.4 | 4.1E-01 | 1.1 | 1.0E+00 |
| TNS1 | **-2.2** | **4.9E-04** | **-2.6** | **1.5E-03** | 1.4 | 5.2E-01 | 1.7 | 1.0E+00 |
| BCL2A1 | **-2.3** | **7.1E-04** | **-2.4** | **1.2E-02** | 1.4 | 5.8E-01 | 1.1 | 1.0E+00 |
| RHCE | **-2.4** | **4.6E-04** | **-2.1** | **4.8E-02** | 1.4 | 5.9E-01 | 1.7 | 1.0E+00 |
| SLC37A3 | **-2.0** | **1.8E-04** | **-2.2** | **1.1E-03** | 1.4 | 3.9E-01 | 1.4 | 1.0E+00 |
| ITGA9 | -1.9 | 5.8E-03 | **-2.1** | **2.4E-02** | 1.3 | 5.4E-01 | 1.2 | 1.0E+00 |
| GCLC | **-2.0** | **3.0E-06** | -1.8 | 4.9E-03 | 1.3 | 3.2E-01 | 1.3 | 1.0E+00 |
| UBE2H | **-2.0** | **1.3E-06** | -1.9 | 1.2E-03 | 1.3 | 2.5E-01 | 1.5 | 8.1E-01 |
| OSBP2 | **-2.6** | **8.5E-05** | **-2.7** | **3.4E-03** | 1.3 | 5.9E-01 | 1.8 | 9.4E-01 |
| AC018755.2 | -1.3 | 3.8E-01 | **-2.5** | **1.9E-02** | 1.3 | 6.4E-01 | -1.2 | 1.0E+00 |
| SIGLEC5 | -1.6 | 4.4E-02 | **-2.0** | **3.3E-02** | 1.3 | 5.3E-01 | 1.1 | 1.0E+00 |
| ATP2C2 | **-2.7** | **1.3E-04** | **-2.9** | **4.5E-03** | 1.3 | 6.6E-01 | 1.3 | 1.0E+00 |
| CA4 | **-2.2** | **6.5E-03** | **-2.5** | **1.9E-02** | 1.3 | 6.7E-01 | 1.1 | 1.0E+00 |
| PLAU | -1.8 | 1.9E-02 | **-2.6** | **1.2E-03** | 1.3 | 6.0E-01 | -1.1 | 1.0E+00 |
| SOCS3 | **-2.7** | **8.5E-06** | **-3.0** | **2.9E-04** | 1.3 | 6.1E-01 | 1.1 | 1.0E+00 |
| AC072022.2 | **-2.1** | **5.3E-03** | **-2.7** | **4.9E-03** | 1.3 | 6.7E-01 | 2.0 | 7.7E-01 |
| SLC7A5 | **-2.3** | **1.2E-03** | **-2.7** | **3.6E-03** | 1.3 | 6.5E-01 | 1.5 | 1.0E+00 |
| GPR146 | -1.7 | 1.2E-02 | **-2.1** | **5.2E-03** | 1.3 | 5.6E-01 | 1.1 | 1.0E+00 |
| ADRB3 | **-2.6** | **3.1E-02** | -2.8 | 9.8E-02 | 1.3 | 8.0E-01 | 2.8 | 8.0E-01 |
| SLFN14 | **-2.4** | **4.2E-06** | -1.9 | 2.3E-02 | 1.3 | 5.6E-01 | 1.4 | 1.0E+00 |
| OPLAH | -1.9 | 1.2E-02 | **-2.6** | **4.7E-03** | 1.3 | 6.7E-01 | 1.0 | 1.0E+00 |
| AL049634.2 | -1.8 | 1.4E-03 | **-2.2** | **1.1E-03** | 1.3 | 5.0E-01 | 1.1 | 1.0E+00 |
| TLR5 | **-2.6** | **5.0E-06** | **-3.0** | **1.7E-04** | 1.3 | 6.2E-01 | 1.3 | 1.0E+00 |
| MAP2K3 | -1.9 | 1.3E-03 | **-2.2** | **2.6E-03** | 1.3 | 5.6E-01 | 1.3 | 1.0E+00 |
| SLC6A8 | **-2.1** | **7.3E-03** | **-2.4** | **1.8E-02** | 1.3 | 6.9E-01 | 1.3 | 1.0E+00 |
| SLC22A4 | **-2.0** | **1.9E-03** | **-2.3** | **6.7E-03** | 1.3 | 6.4E-01 | 1.4 | 1.0E+00 |
| CYSTM1 | **-2.6** | **2.5E-07** | **-2.8** | **5.0E-05** | 1.3 | 6.0E-01 | 1.2 | 1.0E+00 |
| RAD23A | -1.9 | 2.0E-02 | **-2.5** | **7.4E-03** | 1.2 | 7.1E-01 | 1.4 | 1.0E+00 |
| CASP5 | **-2.9** | **1.8E-04** | **-2.5** | **3.1E-02** | 1.2 | 7.6E-01 | 1.7 | 1.0E+00 |
| PYGL | -1.7 | 1.5E-02 | **-2.0** | **2.3E-02** | 1.2 | 6.6E-01 | 1.1 | 1.0E+00 |
| GYG1 | **-2.2** | **3.5E-07** | **-2.2** | **2.7E-04** | 1.2 | 5.5E-01 | -1.0 | 1.0E+00 |
| KLHL2 | -1.9 | 1.5E-03 | **-2.1** | **1.3E-02** | 1.2 | 6.6E-01 | 1.1 | 1.0E+00 |
| GNG10 | -1.9 | 5.2E-03 | **-2.2** | **1.5E-02** | 1.2 | 7.0E-01 | 1.3 | 1.0E+00 |
| KREMEN1 | **-2.9** | **2.5E-05** | **-3.6** | **1.5E-04** | 1.2 | 7.4E-01 | 1.7 | 1.0E+00 |
| NLRC4 | **-2.1** | **1.1E-04** | **-2.2** | **1.8E-03** | 1.2 | 6.4E-01 | 1.0 | 1.0E+00 |
| LMNB1 | **-2.1** | **2.5E-05** | **-2.0** | **3.6E-03** | 1.2 | 6.2E-01 | 1.2 | 1.0E+00 |

| | | | | | | | | |
|---|---|---|---|---|---|---|---|---|
| GCA | **-2.0** | **5.6E-03** | **-2.1** | **3.9E-02** | 1.2 | 7.3E-01 | 1.2 | 1.0E+00 |
| HMBS | **-2.1** | **7.7E-05** | -1.8 | 3.4E-02 | 1.2 | 6.6E-01 | 1.5 | 1.0E+00 |
| MGAM2 | **-3.0** | **1.6E-04** | **-2.8** | **1.1E-02** | 1.2 | 8.0E-01 | 1.7 | 1.0E+00 |
| TGM2 | **-2.3** | **3.5E-07** | **-2.2** | **2.9E-04** | 1.2 | 6.4E-01 | 1.3 | 1.0E+00 |
| MRC2 | **-2.1** | **1.9E-03** | **-2.2** | **2.3E-02** | 1.2 | 7.6E-01 | 1.3 | 1.0E+00 |
| SRPK1 | **-2.0** | **5.9E-05** | **-2.1** | **1.2E-03** | 1.2 | 6.6E-01 | 1.3 | 1.0E+00 |
| FURIN | -1.5 | 4.8E-02 | **-2.0** | **1.3E-02** | 1.2 | 7.0E-01 | 1.2 | 1.0E+00 |
| JAZF1 | **-2.0** | **2.8E-05** | -1.8 | 1.9E-02 | 1.2 | 6.7E-01 | 1.5 | 1.0E+00 |
| LY96 | -1.9 | 8.3E-04 | **-2.1** | **4.2E-03** | 1.2 | 7.2E-01 | 1.2 | 1.0E+00 |
| CD24 | **-2.4** | **5.6E-04** | **-2.1** | **3.9E-02** | 1.2 | 7.9E-01 | -1.2 | 1.0E+00 |
| TFR2 | **-2.1** | **1.6E-05** | **-2.0** | **4.2E-03** | 1.2 | 6.9E-01 | 1.3 | 1.0E+00 |
| MAP3K20 | -1.7 | 1.1E-04 | **-2.0** | **1.5E-04** | 1.2 | 6.2E-01 | 1.0 | 1.0E+00 |
| ADM | **-2.4** | **1.6E-04** | **-2.6** | **3.9E-03** | 1.2 | 7.9E-01 | 1.3 | 1.0E+00 |
| SIPA1L2 | **-2.3** | **1.0E-03** | **-2.2** | **2.9E-02** | 1.2 | 8.0E-01 | -1.0 | 1.0E+00 |
| H1F0 | -1.9 | 7.1E-05 | **-2.0** | **3.1E-03** | 1.2 | 7.0E-01 | -1.6 | 8.2E-01 |
| GRINA | -1.7 | 6.1E-02 | **-2.3** | **2.0E-02** | 1.2 | 8.1E-01 | 1.0 | 1.0E+00 |
| ALDH1A2 | **-2.1** | **2.2E-03** | **-2.2** | **2.1E-02** | 1.2 | 8.1E-01 | 1.2 | 1.0E+00 |
| ACSL1 | **-2.4** | **9.9E-04** | **-2.3** | **3.1E-02** | 1.2 | 8.3E-01 | 1.1 | 1.0E+00 |
| KIAA0825 | -1.9 | 1.8E-04 | **-2.0** | **4.3E-03** | 1.2 | 7.3E-01 | 1.3 | 1.0E+00 |
| LILRA5 | -1.9 | 3.5E-03 | **-2.3** | **5.3E-03** | 1.2 | 7.9E-01 | 1.2 | 1.0E+00 |
| WDFY3 | **-2.0** | **1.1E-03** | **-2.0** | **2.1E-02** | 1.1 | 8.0E-01 | 1.2 | 1.0E+00 |
| PLSCR1 | **-2.1** | **4.4E-04** | -1.8 | 6.3E-02 | 1.1 | 8.1E-01 | 1.1 | 1.0E+00 |
| ZNF438 | -1.9 | 1.7E-04 | **-2.1** | **2.2E-03** | 1.1 | 7.6E-01 | 1.2 | 1.0E+00 |
| SIRPD | -1.7 | 3.9E-03 | **-2.1** | **1.8E-03** | 1.1 | 7.8E-01 | -1.1 | 1.0E+00 |
| KCNJ15 | **-2.2** | **4.3E-04** | **-2.1** | **1.9E-02** | 1.1 | 8.3E-01 | 1.3 | 1.0E+00 |
| OSM | **-2.8** | **6.9E-07** | **-3.0** | **8.4E-05** | 1.1 | 8.3E-01 | 1.2 | 1.0E+00 |
| HCAR3 | **-2.0** | **2.9E-04** | -1.7 | 6.3E-02 | 1.1 | 8.0E-01 | 1.1 | 1.0E+00 |
| SEMA4A | -1.7 | 2.1E-03 | **-2.0** | **7.5E-04** | 1.1 | 7.7E-01 | 1.0 | 1.0E+00 |
| LILRA6 | -1.7 | 8.3E-03 | **-2.2** | **3.0E-03** | 1.1 | 8.2E-01 | -1.1 | 1.0E+00 |
| DOCK4 | **-2.4** | **2.6E-05** | **-2.3** | **4.3E-03** | 1.1 | 8.5E-01 | -1.1 | 1.0E+00 |
| NAIP | **-2.3** | **7.9E-07** | **-2.5** | **9.3E-05** | 1.1 | 8.3E-01 | -1.4 | 1.0E+00 |
| CEACAM1 | **-2.3** | **2.0E-03** | -1.9 | 9.9E-02 | 1.1 | 8.8E-01 | 1.2 | 1.0E+00 |
| F5 | **-2.3** | **6.4E-06** | **-2.5** | **3.3E-04** | 1.1 | 8.5E-01 | 1.3 | 1.0E+00 |
| MCTP2 | **-2.0** | **2.8E-04** | -1.9 | 2.3E-02 | 1.1 | 8.5E-01 | 1.0 | 1.0E+00 |
| IL4R | **-2.3** | **2.5E-07** | **-2.4** | **8.4E-05** | 1.1 | 8.3E-01 | 1.3 | 1.0E+00 |
| RAB3IL1 | **-2.0** | **1.5E-02** | -2.1 | 5.3E-02 | 1.1 | 9.0E-01 | 1.5 | 1.0E+00 |
| MGAM | **-2.3** | **2.1E-03** | **-2.4** | **2.2E-02** | 1.1 | 9.0E-01 | 1.4 | 1.0E+00 |
| C3orf86 | -1.9 | 6.7E-03 | **-2.1** | **3.2E-02** | 1.1 | 8.9E-01 | 1.2 | 1.0E+00 |
| S100P | **-2.0** | **3.4E-02** | -2.3 | 5.7E-02 | 1.1 | 9.2E-01 | -1.4 | 1.0E+00 |
| HORMAD1 | -1.8 | 4.6E-03 | **-2.1** | **6.4E-03** | 1.1 | 8.8E-01 | 1.4 | 1.0E+00 |
| LRG1 | **-2.0** | **2.4E-03** | **-2.0** | **3.6E-02** | 1.1 | 8.9E-01 | -1.0 | 1.0E+00 |
| NLRP6 | -1.4 | 1.7E-01 | **-2.0** | **3.3E-02** | 1.1 | 9.1E-01 | 1.2 | 1.0E+00 |
| BCAM | **-2.6** | **7.0E-05** | -2.0 | 6.9E-02 | 1.1 | 9.2E-01 | 1.5 | 1.0E+00 |

| | | | | | | | | |
|---|---|---|---|---|---|---|---|---|
| PLSCR2 | **-2.5** | **3.5E-06** | **-2.0** | **2.2E-02** | 1.1 | 9.1E-01 | 1.1 | 1.0E+00 |
| SEPTIN14 | **-2.4** | **2.0E-06** | **-2.1** | **4.2E-03** | 1.1 | 9.0E-01 | 1.8 | 5.6E-01 |
| GABRR2 | **-2.2** | **1.4E-05** | **-2.0** | **5.9E-03** | 1.1 | 9.1E-01 | -1.1 | 1.0E+00 |
| PROK2 | **-2.2** | **8.2E-04** | **-2.1** | **3.4E-02** | 1.1 | 9.3E-01 | 1.2 | 1.0E+00 |
| FAM157B | **-2.3** | **3.6E-04** | **-2.1** | **2.8E-02** | 1.0 | 9.5E-01 | 1.5 | 1.0E+00 |
| FLOT2 | -1.8 | 3.0E-02 | **-2.2** | **1.9E-02** | 1.0 | 9.6E-01 | 1.1 | 1.0E+00 |
| KANK2 | -1.9 | 2.7E-03 | **-2.2** | **3.4E-03** | 1.0 | 9.6E-01 | -1.0 | 1.0E+00 |
| CRIP2 | **2.0** | **2.8E-04** | 1.6 | 9.6E-02 | 1.0 | 9.5E-01 | -1.5 | 1.0E+00 |
| SERPINA1 | -1.8 | 2.0E-02 | **-2.0** | **3.3E-02** | 1.0 | 9.7E-01 | 1.2 | 1.0E+00 |
| DYSF | **-2.1** | **5.1E-03** | **-2.5** | **7.2E-03** | 1.0 | 9.8E-01 | 1.2 | 1.0E+00 |
| CREB5 | **-2.0** | **6.6E-04** | **-2.0** | **1.2E-02** | 1.0 | 9.7E-01 | 1.4 | 1.0E+00 |
| OCLN | **-2.0** | **1.7E-03** | -1.8 | 9.8E-02 | 1.0 | 9.8E-01 | 1.1 | 1.0E+00 |
| AQP9 | **-2.0** | **1.8E-03** | -1.9 | 4.1E-02 | 1.0 | 9.8E-01 | 1.1 | 1.0E+00 |
| DNAJC25-GNG10 | **-2.0** | **3.6E-04** | -1.9 | 2.4E-02 | 1.0 | 9.8E-01 | 1.2 | 1.0E+00 |
| DOK3 | -1.7 | 3.5E-02 | **-2.1** | **2.2E-02** | 1.0 | 9.9E-01 | 1.1 | 1.0E+00 |
| CR1 | **-2.3** | **4.7E-05** | **-2.4** | **2.9E-03** | 1.0 | 9.9E-01 | 1.0 | 1.0E+00 |
| GALNT14 | **-4.0** | **9.3E-08** | **-4.1** | **9.4E-05** | 1.0 | 1.0E+00 | -1.1 | 1.0E+00 |
| GK | **-2.1** | **7.9E-05** | -1.8 | 3.8E-02 | 1.0 | 1.0E+00 | 1.2 | 1.0E+00 |
| LRRN1 | **-2.0** | **2.1E-03** | **-2.3** | **5.5E-03** | 1.0 | 1.0E+00 | 1.6 | 1.0E+00 |
| PLB1 | **-2.2** | **2.6E-05** | **-2.3** | **8.5E-04** | 1.0 | 1.0E+00 | 1.4 | 1.0E+00 |
| TECPR2 | -1.9 | 5.2E-04 | **-2.0** | **6.8E-03** | -1.0 | 1.0E+00 | 1.2 | 1.0E+00 |
| PGS1 | **-2.2** | **1.9E-06** | **-2.1** | **2.7E-03** | -1.0 | 9.8E-01 | 1.1 | 1.0E+00 |
| BASP1 | **-2.0** | **1.9E-03** | **-2.1** | **2.3E-02** | -1.0 | 9.8E-01 | 1.3 | 1.0E+00 |
| PFKFB3 | **-2.9** | **1.2E-07** | **-2.7** | **3.3E-04** | -1.0 | 9.8E-01 | 1.2 | 1.0E+00 |
| IL1B | **-2.2** | **2.6E-05** | **-2.0** | **1.1E-02** | -1.0 | 9.8E-01 | 1.3 | 1.0E+00 |
| TRPM6 | **-2.3** | **2.8E-06** | **-2.1** | **2.6E-03** | -1.0 | 9.7E-01 | 1.3 | 1.0E+00 |
| ANKRD40CL | **-2.0** | **1.5E-02** | -1.9 | 1.1E-01 | -1.0 | 9.7E-01 | 1.7 | 1.0E+00 |
| USB1 | **-2.0** | **7.7E-05** | **-2.0** | **6.6E-03** | -1.1 | 9.1E-01 | 1.1 | 1.0E+00 |
| DHRS13 | **-2.3** | **7.9E-06** | -1.9 | 2.3E-02 | -1.1 | 9.1E-01 | 1.2 | 1.0E+00 |
| AC011498.4 | **-2.1** | **4.9E-04** | **-2.3** | **3.0E-03** | -1.1 | 9.0E-01 | 1.3 | 1.0E+00 |
| HRH2 | **-2.0** | **7.1E-04** | -1.8 | 4.7E-02 | -1.1 | 8.9E-01 | 1.2 | 1.0E+00 |
| IL1R1 | **-2.2** | **4.6E-03** | **-2.2** | **4.9E-02** | -1.1 | 9.0E-01 | 1.0 | 1.0E+00 |
| PADI2 | -1.7 | 4.2E-02 | **-2.0** | **3.8E-02** | -1.1 | 8.7E-01 | -1.0 | 1.0E+00 |
| ITGB4 | **-3.4** | **3.4E-09** | **-4.4** | **1.9E-09** | -1.1 | 8.7E-01 | 1.1 | 1.0E+00 |
| CST7 | **-2.3** | **3.6E-04** | **-2.8** | **1.1E-03** | -1.1 | 8.6E-01 | -1.2 | 1.0E+00 |
| MED12L | **-2.1** | **1.9E-05** | -1.4 | 2.2E-01 | -1.1 | 8.2E-01 | -1.1 | 1.0E+00 |
| LIMK2 | **-2.1** | **3.0E-04** | -1.9 | 3.4E-02 | -1.1 | 8.3E-01 | 1.2 | 1.0E+00 |
| BCL6 | **-2.2** | **1.3E-03** | **-2.2** | **3.4E-02** | -1.1 | 8.6E-01 | 1.4 | 1.0E+00 |
| ST3GAL4 | **-2.3** | **1.5E-06** | **-2.2** | **1.5E-03** | -1.1 | 7.7E-01 | 1.1 | 1.0E+00 |
| AC099489.1 | -1.8 | 2.2E-02 | **-2.0** | **4.2E-02** | -1.1 | 8.3E-01 | 1.2 | 1.0E+00 |
| FGFBP2 | 1.3 | 1.7E-01 | **2.0** | **4.5E-03** | -1.1 | 7.5E-01 | 1.0 | 1.0E+00 |
| ALPL | **-3.6** | **1.8E-05** | **-3.7** | **1.5E-03** | -1.2 | 8.3E-01 | 1.4 | 1.0E+00 |

| | | | | | | | | |
|---|---|---|---|---|---|---|---|---|
| MRVI1 | **-2.0** | **1.3E-03** | -1.9 | 4.6E-02 | -1.2 | 7.5E-01 | 1.2 | 1.0E+00 |
| IL18RAP | -1.9 | 3.9E-03 | **-2.0** | **2.9E-02** | -1.2 | 7.4E-01 | -1.2 | 1.0E+00 |
| TIGD3 | **2.0** | **1.4E-03** | 1.6 | 1.6E-01 | -1.2 | 7.2E-01 | -1.1 | 1.0E+00 |
| DUSP2 | 1.5 | 6.0E-02 | **2.0** | **5.5E-03** | -1.2 | 6.8E-01 | -1.3 | 1.0E+00 |
| DSC2 | **-2.6** | **5.0E-06** | **-2.4** | **3.1E-03** | -1.2 | 7.3E-01 | 1.0 | 1.0E+00 |
| ADGRG3 | **-2.5** | **7.3E-04** | **-2.5** | **1.8E-02** | -1.2 | 7.6E-01 | 1.2 | 1.0E+00 |
| CDH19 | **-2.1** | **4.7E-03** | -1.7 | 1.6E-01 | -1.2 | 7.3E-01 | 1.6 | 1.0E+00 |
| HEPH | **-2.0** | **1.4E-02** | -2.0 | 6.7E-02 | -1.2 | 7.3E-01 | -1.2 | 1.0E+00 |
| FCER1A | **2.9** | **4.6E-04** | **3.7** | **1.2E-03** | -1.2 | 7.6E-01 | 1.0 | 1.0E+00 |
| C1orf21 | 1.2 | 2.6E-01 | **2.0** | **1.0E-03** | -1.2 | 4.8E-01 | -1.1 | 1.0E+00 |
| FAM157A | **-2.6** | **2.0E-03** | -2.3 | 5.6E-02 | -1.3 | 7.4E-01 | 1.3 | 1.0E+00 |
| PLIN4 | **-2.3** | **5.6E-03** | **-2.3** | **4.8E-02** | -1.3 | 6.8E-01 | 1.4 | 1.0E+00 |
| TTC26 | **-2.2** | **3.8E-04** | -1.6 | 1.6E-01 | -1.3 | 5.5E-01 | 1.1 | 1.0E+00 |
| AKAP12 | **2.4** | **8.0E-03** | **4.2** | **8.2E-04** | -1.3 | 6.9E-01 | 1.1 | 1.0E+00 |
| CACNB4 | 1.7 | 4.9E-04 | **2.1** | **1.7E-04** | -1.4 | 2.1E-01 | -1.5 | 8.8E-01 |
| ITGB8 | 1.7 | 5.9E-02 | **2.5** | **9.4E-03** | -1.4 | 5.3E-01 | 1.0 | 1.0E+00 |
| S1PR5 | 1.2 | 3.3E-01 | **2.0** | **1.6E-03** | -1.4 | 2.4E-01 | -1.3 | 1.0E+00 |
| DOK4 | **-2.5** | **1.8E-07** | **-2.2** | **1.2E-03** | -1.4 | 3.0E-01 | 1.1 | 1.0E+00 |
| LPO | **-2.2** | **8.3E-04** | -1.5 | 2.9E-01 | -1.4 | 4.1E-01 | 1.3 | 1.0E+00 |
| LYVE1 | **-2.3** | **7.3E-04** | -1.9 | 6.2E-02 | -1.4 | 4.4E-01 | 1.5 | 1.0E+00 |
| TRIM9 | **-3.1** | **4.3E-06** | **-2.9** | **1.8E-03** | -1.4 | 4.7E-01 | 1.3 | 1.0E+00 |
| KAZN | **-2.5** | **5.6E-07** | **-2.7** | **3.0E-05** | -1.4 | 2.6E-01 | 1.5 | 1.0E+00 |
| MS4A2 | 1.7 | 1.0E-01 | **3.2** | **1.5E-03** | -1.5 | 4.9E-01 | -1.2 | 1.0E+00 |
| COLGALT2 | 1.4 | 3.5E-02 | **2.0** | **3.5E-04** | -1.5 | 6.3E-02 | -1.2 | 1.0E+00 |
| NPB | **-2.0** | **3.9E-02** | -1.3 | 6.4E-01 | -1.5 | 5.2E-01 | -1.0 | 1.0E+00 |
| HDC | **4.0** | **6.6E-04** | **8.9** | **5.7E-05** | -1.5 | 6.4E-01 | -1.4 | 1.0E+00 |
| PCSK5 | **2.2** | **2.0E-02** | **2.7** | **2.8E-02** | -1.7 | 3.2E-01 | -3.3 | 2.2E-01 |
| SLC45A3 | 1.9 | 6.2E-02 | **3.3** | **3.7E-03** | -1.8 | 2.4E-01 | -1.1 | 1.0E+00 |
| HRH4 | 1.4 | 9.8E-02 | **2.1** | **3.4E-03** | -1.9 | 6.3E-03 | 1.1 | 1.0E+00 |
| GATA2 | 2.6 | 5.7E-02 | **5.9** | **3.0E-03** | -2.1 | 3.1E-01 | -1.4 | 1.0E+00 |
